# Fecal Microbiota Transplantation Improves Cognitive Function of a Mouse Model of Alzheimer's Disease

**DOI:** 10.1111/cns.70259

**Published:** 2025-02-17

**Authors:** Xueqin Jiang, Yu Zheng, Huaiqing Sun, Yini Dang, Mengmei Yin, Ming Xiao, Ting Wu

**Affiliations:** ^1^ Department of Neurology The First Affiliated Hospital of Nanjing Medical University Nanjing China; ^2^ Jiangsu Key Laboratory of Neurodegeneration Nanjing Medical University Nanjing China; ^3^ Department of Rehabilitation Medicine The First Affiliated Hospital of Nanjing Medical University Nanjing China; ^4^ Department of Gastroenterology The First Affiliated Hospital of Nanjing Medical University Nanjing China; ^5^ Brain Institute, Nanjing Brain Hospital Nanjing Medical University Nanjing China

**Keywords:** β‐amyloid, Alzheimer's disease, fecal microbiota transplantation, gut microbiota, lipopolysaccharide

## Abstract

**Background:**

A growing body of evidence suggests a link between the gut microbiota and Alzheimer's disease (AD), although the underlying mechanisms remain elusive. This study aimed to investigate the impact of fecal microbiota transplantation (FMT) on cognitive function in a mouse model of AD.

**Methods:**

Four‐month‐old 5 × FAD (familial Alzheimer's disease) mice underwent antibiotic treatment to deplete their native gut microbiota. Subsequently, they received FMT either weekly or every other day. After 8 weeks, cognitive function and β‐amyloid (Aβ) load were assessed through behavioral testing and pathological analysis, respectively. The composition of the gut microbiota was analyzed using 16S rRNA sequencing.

**Results:**

Initial weekly FMT failed to alleviate memory deficits or reduce brain Aβ pathology in 5 × FAD mice. In contrast, FMT administered every other day effectively restored gut dysbiosis in 5 × FAD mice and decreased Aβ pathology and lipopolysaccharide levels in the colon and hippocampus. Mechanistically, FMT reduced the expression of amyloid β precursor protein, β‐site APP cleaving enzyme 1, and presenilin‐1, potentially by inhibiting the Toll‐like receptor 4/inhibitor of kappa B kinase β/nuclear factor kappa‐B signaling pathway. However, the cognitive benefits of FMT on 5 × FAD mice diminished over time.

**Conclusion:**

These findings demonstrate the dose‐ and time‐dependent efficacy of FMT in mitigating AD‐like pathology, underscoring the potential of targeting the gut microbiota for AD treatment.

## Graphical abstract

Fecal microbiota transplantation modified the gut microbiota composition in 5 × FAD mice, resulting in decreased gut microbial‐derived lipopolysaccharide. This alteration subsequently enhanced the cognitive function of 5 × FAD mice by inhibiting the hippocampal TLR4‐IKKβ‐NF‐κB signaling pathway.

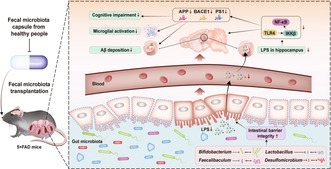

## 1 Introduction

Alzheimer's disease (AD) is the leading cause of dementia among older individuals [1], characterized by senile plaques and neurofibrillary tangles resulting from abnormal Aβ aggregation and tau protein phosphorylation [2]. Aβ peptides are generated through the cleavage of amyloid β precursor protein (APP) by β‐ and γ‐secretase [3]. Multiple pathways and mechanisms, including enzymatic degradation, microglial phagocytosis, blood–brain barrier (BBB) transport, and glymphatic drainage, contribute to the clearance of Aβ from the brain [4]. An imbalance between the production and clearance of Aβ leads to its aberrant deposition, thereby exacerbating neurodegenerative processes [5].

The microbiome‐gut‐brain axis, encompassing the immune system, neuroendocrine system, enteric nervous system, and gut microbial metabolites, constitutes a complex bidirectional communication network between the gut and the brain [6]. This axis is implicated in AD, although its precise pathophysiology remains elusive. Compared to healthy controls, the gut microbiota of AD patients exhibits variations in taxonomic composition [7]. Several studies have documented a decreased relative abundance of *Firmicutes* and *Bifidobacterium*, coupled with increased *Proteobacteria* and *Bacteroidetes*, in AD patients [8, 9, 10]. Similarly, alterations in the gut microbiota are observed in AD mouse models, such as 5 × FAD mice [11], APP/PS1 mice [12, 13], and TgCRND8 mice [14]. Interestingly, modulation of gut microbiota composition through long‐term antibiotic therapy attenuates amyloidogenic processes in 5 × FAD transgenic mice [11]. Conversely, healthy adult rats develop memory deficits following gut microbiome transplantation from AD patients [15]. These findings underscore the involvement of the gut microbiota in the development and progression of AD.

Alterations in the gut microbiome can influence intestinal permeability [16]. Gut microbes produce various innate immune stimulants, including lipopolysaccharide (LPS). Notably, gut microbiota‐derived LPS, a potent activator of toll‐like receptor 4 (TLR4) on microglia, can penetrate the compromised BBB, activating the TLR4/NF‐κB signaling pathway. This, in turn, initiates a cascade of inflammatory responses within the brain, ultimately exacerbating AD pathology [17, 18, 19]. It has been reported that nuclear factor kappa‐B (NF‐κB) may be crucial in regulating Aβ metabolism [20, 21]. *Bifidobacterium longum* , a probiotic strain, suppresses LPS‐induced NF‐κB activation, reversing cognitive deficits in 5 × FAD and aged mice [22]. Overall, intestinal inflammation may exacerbate AD progression through these mechanisms.

Several studies conducted using AD mouse models have demonstrated the potential of FMT in ameliorating AD‐like pathology and cognitive deficits [23, 24, 25]. However, it remains unclear whether colonizing 5 × FAD mice with fecal microbiota from healthy human donors can alleviate their cognitive impairments. To this end, in this study, we administered fecal microbiota capsules derived from healthy human donors to 5 × FAD mice. We assessed the impact of FMT on their gut microbiota composition, AD‐like pathology, and cognitive function and further elucidated the underlying mechanisms.

## 2 Materials and Methods

### 2.1 Animals and Experimental Design

C57BL/6J mice, including both wild‐type (WT) and 5 × FAD [APP K670N/M671L (Swedish) + I716V (Florida) + V717I (London) and PS1 M146L + L286V] [26], were obtained from Jackson Laboratory. These mice were housed at Nanjing Medical University's Experimental Animal Center under standard environmental conditions, with a 12‐h light/dark cycle and access to sterile food and water. All animal experiments were approved by the Animal Ethical and Welfare Committee of Nanjing Medical University (No. IACUC‐2207023). Furthermore, informed consent was obtained from healthy human donors, and ethical approval (2022‐SR‐419) was received from the First Affiliated Hospital of Nanjing Medical University for using these fecal microbiota capsules in clinical research.

Before the experiment, a bacterial solution with a concentration of 5.0 × 10^9^ colony‐forming units (CFU)/mL was prepared by dissolving the contents of fecal microbiota capsules derived from healthy humans (Precision Gene Technology Co. Ltd., Jiangxi, China) in sterile 0.01 M phosphate‐buffered saline (PBS, pH 7.4). Detailed information on these fecal microbiota capsules is listed in Table S1. Three groups of 4‐month‐old mice (female: male = 1:2) were established: a WT group, a 5 × FAD group, and a 5 × FAD‐FMT group. Following established protocols with minor modifications [27, 28], mice in the 5 × FAD‐FMT group were administered a cocktail of ampicillin (1 g/L; Sigma‐Aldrich), streptomycin (5 g/L, MCE), and colistin (1 g/L; Sigma‐Aldrich) in drinking water for 1 week to eliminate their endogenous microbiota. Subsequently, 5 × FAD‐FMT mice received an 8‐week gavage regimen, with each dose consisting of 0.2 mL of a fresh fecal microbiota solution containing 1.0 × 10^9^ CFU. Concurrently, mice in the 5 × FAD and WT groups were administered a comparable volume of PBS via gavage for the same duration. All mice were maintained under identical feeding and environmental conditions to ensure consistency. Their body weights were monitored weekly throughout the treatment period.

**FIGURE 1:**
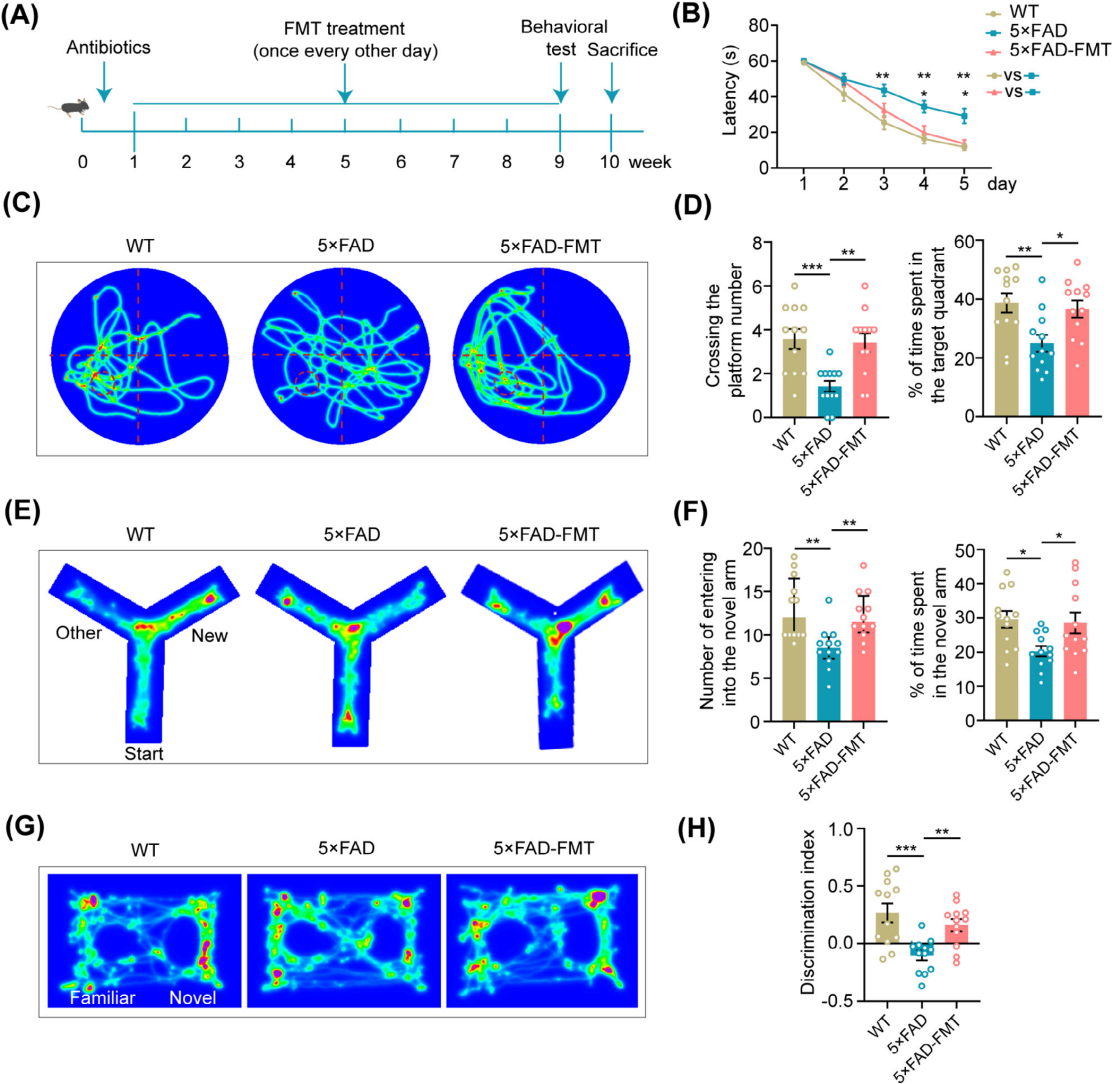
Cognitive improvements in 5 × FAD mice following FMT. (A) Diagrammatic illustration of the experimental process. Mice underwent antibiotic treatment, FMT (administered every other day for 8 weeks), behavioral tests, and sacrifices. (B) Statistical analysis of the latency for each mouse to reach the platform during the 5‐day training period in the Morris water maze. (C) Representative trajectories of mice on the sixth day (probe trial) of the Morris water maze. (D) Statistical analysis of the number of platform crossings and the percentage of time spent in the target quadrant during the Morris water maze probe trial. (E) Movement tracks of mice in the testing phase of the Y‐maze test. (F) Statistical analysis of the number of entries into the novel arm and the duration of exploration in the Y‐maze. (G) Movement tracks of mice in the novel object recognition test. (H) Statistical analysis of the discrimination index for novel objects among the mice. *n* = 12 per group. Significance was evaluated using repeated measures two‐way ANOVA with Tukey's post hoc test for the latency data in (B), the Kruskal‐Wallis test with Dunn's post hoc analysis for the number of entries into the novel arm in (F), and one‐way ANOVA with Dunnett's post hoc test for all other data. *p < 0.05, **p < 0.01, ***p < 0.001.

This study involved three consecutive rounds of animal experiments. In the first round, FMT was administered once a week to one group of 13 mice. For the second and third rounds, each round included 36 mice (*n* = 12 per group), with one group receiving FMT every other day. Following the 8‐week FMT regimen, all mice in the first and second rounds were immediately subjected to behavioral tests to assess their cognitive function. In contrast, mice in the third round underwent behavioral testing after a 4‐week observation period post‐FMT treatment. Seven mice from each group in the second round were randomly selected for 16S rRNA sequencing. The aforementioned animal experimental design is illustrated in Figure S1A, Figure 1A, and Figure S7A.

### 2.2 Supplementary LPS Experiment

Four‐month‐old 5 × FAD mice were randomly divided into four groups: a control group (CON), an FMT group, an LPS group, and an FMT + LPS group, with four mice in each group. Following established protocols, mice in the FMT and FMT + LPS groups underwent FMT every other day for 8 weeks after a 1‐week course of antibiotic‐containing drinking water. Starting from the sixth week, mice in the LPS and FMT + LPS groups received intraperitoneal injections of LPS (from *E. coli* , serotype 055:B5; Sigma‐Aldrich, St. Louis, MO, USA) at a dose of 250 μg/kg body weight, twice weekly, for 4 weeks.

### 2.3 Fecal Sample Collection

As previously described [29], each mouse was placed in a sterilized cage on the morning following the final gavage intervention. Fresh fecal pellets (six to eight per mouse) were promptly collected and transferred to a pre‐sterilized Eppendorf tube to prevent urine contamination. The fecal samples were immediately frozen and stored at −80°C for microbiome analysis.

### 2.4 Morris Water Maze

According to a previously established protocol [30], we utilized the Morris water maze test to assess the mice's spatial memory capabilities. The maze was filled with an opaque mixture of water and milk to obscure the submerged platform, maintaining a temperature range of 19°C–20°C. It was divided into four quadrants, each featuring distinct colored geometric shapes to facilitate navigation. A submerged platform was located in one of the quadrants. The experiment comprised a 5‐day training session followed by a 1‐day testing phase. During the training period, each mouse underwent four trials per day, each lasting 1 min, in which they were trained to locate the hidden platform. If a mouse failed to find the platform within 1 min, it was guided to the platform and allowed to remain there for 15 s. On the sixth day, the hidden platform was removed, and each mouse was released into the water from the quadrant opposite the platform, allowing them to swim freely for 1 min. The latency to reach the platform, the number of platform crossings, and the percentage of time spent exploring the target quadrant were analyzed.

### 2.5 Y‐Maze Test

The Y‐maze test was employed to assess the mice's transient spatial memory [31]. Two distinct phases, adaptation and testing, were conducted using a “Y”‐shaped maze with three identical arms connected at a uniform angle. During the adaptation phase, mice were confined to explore two arms for 5 min, with a movable barrier blocking access to the third arm. Subsequently, during the testing phase, the barrier was removed, allowing the mice to freely explore all three arms for an additional 5 min. The proportion of time spent in the novel arm and the number of entries into it was recorded.

### 2.6 Novel Object Recognition Test

The novel object recognition test is a reliable measure to assess the short‐term memory capabilities of mice [32]. The experimental apparatus consisted of a rectangular box (50 × 35 cm). Initially, two identical plastic objects were placed symmetrically within the box, each positioned 5 cm from the nearest wall. Subsequently, mice were placed in the center of the box between the two objects and allowed to explore freely for 5 min. After a 2‐h interval, one of the familiar objects was replaced with a novel object of a different shape. The mouse was then reintroduced to the box and allowed to explore the objects for 5 min, with the time spent sniffing or touching each object being recorded. *T*_1_ and *T*_2_ represented the exploration times for the familiar and novel objects, respectively. The discrimination index was calculated as (*T*_2_ − *T*_1_)/(*T*_1_ + *T*_2_).

Mouse movements were captured using video tracking software (Beijing Sunny Instruments Co. Ltd., China) throughout the behavioral tests. The room was dimly lit to maintain the mice's comfort. The equipment was sanitized with 75% alcohol before each test session. Two experimenters conducted the tests in a double‐blind manner.

### 2.7 Animal Sampling and Tissue Preparation

After behavioral testing, mice were anesthetized, and their brain and colon tissues were immediately isolated following transcardial perfusion with pre‐cooled 0.01 M PBS (pH 7.4) for 5 min. One hemisphere of each brain was immersed in 4% paraformaldehyde for overnight fixation at 4°C, followed by sequential dehydration in 20% and 30% sucrose solutions at 4°C for 3 days. Brain tissues were then embedded in an optimal cutting temperature compound and sectioned into 20‐μm‐thick coronal brain slices using a vibrating microtome (VT1200; Leica, Solms, Germany). Concurrently, the distal 1‐cm segment of the colon was preserved overnight at 4°C in 4% paraformaldehyde. Colon tissues underwent graded ethanol dehydration, xylene clearing, and paraffin embedding, followed by sectioning into 5‐μm‐thick paraffin slices using a sliding microtome (SM2000R; Leica, Solms, Germany). These slices were incubated at 37°C overnight and then stored at room temperature. The remaining brain and colon tissues were immediately frozen and stored at −80°C for subsequent analyses, including Western blotting (WB), enzyme‐linked immunosorbent assay (ELISA), and quantitative real‐time polymerase chain reaction (qRT‐PCR).

### 2.8 Immunofluorescence Assay

As previously described [33], tissue sections from the brains and colons were blocked with a 0.3% PBS‐Triton X‐100 solution containing 5% bovine serum albumin for 1 h at room temperature. Subsequently, the sections were incubated with appropriately diluted primary antibodies (Table S2) overnight at 4°C. After washing with 0.01 M PBS (pH 7.4), the sections were incubated with appropriate secondary antibodies (Table S2) in the dark for 2 h at room temperature. Following a staining step with a 1:1000 dilution of 4′,6‐diamidino‐2‐phenylindole (DAPI) (Invitrogen; Cat. # D21490) for 8 min, the sections were mounted with glass coverslips and imaged under a fluorescence microscope. Quantitative analysis of positively stained areas was performed using ImageJ (National Institutes of Health, USA).

### 2.9 Skeleton Analysis of Microglia Morphology

Based on the staining results of ionized calcium‐binding adapter molecule 1 (Iba1), we analyzed microglia morphology using ImageJ, following the method described by Young and Morrison [34]. In 8‐bit binary images, microglia were skeletonized to quantitatively assess the number of endpoints and branches, as well as the total length of processes.

### 2.10 Immunohistochemistry Assay

According to a previously established protocol [35], colon tissue samples were first deparaffinized and hydrated. Antigen retrieval was then achieved through heat‐induced treatment in a citric acid buffer (pH 6.0). Endogenous peroxidase was inactivated by incubation with 3% H_2_O_2_ for 20 min. The 6E10 primary antibody (Table S2) was then incubated overnight at 4°C to target the specific antigen. The sections were thoroughly washed with 0.01 M PBS (pH 7.4) before exposure to a secondary goat anti‐mouse IgG antibody conjugated to horseradish peroxidase (Table S2) for 1 h at 37°C. All sections were visualized using a freshly prepared 3,3′‐diaminobenzidine (DAB) solution, and some were counterstained with hematoxylin.

### 2.11 Western Blotting

Sodium dodecyl sulfate‐polyacrylamide gel electrophoresis (SDS‐PAGE) was employed for protein electrophoresis with denatured protein samples from the colon and hippocampus. After transferring the proteins to polyvinylidene fluoride (PVDF) membranes (Millipore, USA), the samples were blocked with 5% skim milk for 1 h at room temperature and then incubated with primary antibodies (Table S2) overnight at 4°C. On the second day, the membranes were washed three times with tris‐buffered saline with Tween 20 (TBST, pH 7.5, 10 mM Tris–HCl, 150 mM NaCl, and 0.1% Tween 20) before being incubated with secondary antibodies conjugated with horseradish peroxidase (Table S2) for 1 h at room temperature. A chemiluminescence imager (Image Quant LAS 4000 mini, version 1.2) was used to visualize the membranes after they were rewashed with TBST. The protein gray value was quantified using ImageJ (National Institutes of Health, USA) with glyceraldehyde‐3‐phosphate dehydrogenase (GAPDH) utilized as the internal reference for protein expression analysis.

### 2.12 qRT‐PCR

Total RNA was extracted from the hippocampus and colon using RNAiso Plus (Takara, Japan) and quantified using a Nanodrop 2000 (Thermo Fisher, USA). mRNA was reverse transcribed into cDNA using HiScript III RT SuperMix for qPCR (+gDNA wiper) (R323; Vazyme Biotech Co. Ltd., USA). Real‐time quantitative PCR (qRT‐PCR) was performed using the ABI 7300 Fast Real‐Time PCR System (Applied Biosystems, USA) with SYBR Green qPCR Master Mix (Q712; Vazyme Biotech Co. Ltd., USA). The relative mRNA levels of target genes were calculated using the $2^{-\Delta \Delta C_{t}}$ method, with GAPDH as the internal reference gene [32]. The following primers were synthesized by Sangon Biotech Co. Ltd. (Shanghai, China): interleukin‐1β (IL‐1β): forward, 5′‐CTCGCAGCAGCACATCAACAAG‐3′, reverse, 5′‐CCACGGGAAAGACACAGGTAGC‐3′; interleukin‐6 (IL‐6): forward, 5′‐GGAGCCCACCAAGAACGATAGTC‐3′, reverse, 5′‐TCACCAGCATCAGTCCCAAGAAG‐3′; tumor necrosis factor‐α (TNF‐α): forward, 5′‐ACGCTCTTCTGTCTACTGAACTTCG‐3′, reverse, 5′‐TGGTTTGTGAGTGTGAGGGTCTG‐3′; GAPDH: forward, 5′‐AGAAGGTGGTGAAGCAGGCATC‐3′, reverse, 5′‐CGAAGGTGGAAGAGTGGGAGTTG‐3′.

### 2.13 ELISA

To homogenize the hippocampus and colon tissues, ice‐cold radioimmunoprecipitation assay (RIPA) lysis buffer (Beyotime, Cat. # P0013B) containing phosphatase inhibitors (Roche, Cat. # 04906845001) and protease inhibitors (Beyotime, Cat. # st506) was added to the samples. The samples were then centrifuged at 12,000 *g* for 30 min. The supernatants containing the solubilized proteins were carefully aspirated and collected for measurement. Following the manufacturer's instructions, the ELISA kit (Xinfan Biotechnology Co. Ltd., Nanjing, China) was then used to measure the LPS levels in the colon, serum, and hippocampus.

### 2.14 HE Staining

Colon paraffin sections were deparaffinized and hydrated, followed by hematoxylin and eosin (H&E) staining using a staining kit (Beijing Solarbio Technology Co. Ltd., Beijing, China) according to the manufacturer's protocol. Histopathological changes in the mouse colon tissues were observed under a microscope (Olympus, Japan). The histologic score was determined by summing the scores for tissue damage and inflammatory cell infiltration, following established scoring criteria [29]. Tissue damage was graded on a four‐point scale: 0, no damage; 1, lymphoepithelial lesions; 2, focal ulcers; and 3, extensive mucosal damage. Similarly, inflammatory cell infiltration was scored on a four‐point scale: 0, few inflammatory cells in the lamina propria; 1, increased inflammatory cell infiltration in the lamina propria; 2, infiltration into the submucosa; and 3, transmural infiltration of inflammatory cells.

### 2.15 Thioflavin‐S Staining

Brain sections at identical levels were selected and stained with 1% thioflavin‐S (Sigma‐Aldrich, Cat. # T1892) for 5 min. Subsequently, the slices were differentiated with 70% alcohol for 5 min, washed with 0.01 M PBS (pH 7.4), and sealed with buffered PBS/glycerol. The stained brain sections were then examined under 488 nm excitation light, and micrographs were captured and archived for subsequent analysis.

### 2.16 16S rRNA Sequencing

Microbial DNA was extracted from the obtained mouse feces and quantified using a Nanodrop spectrophotometer. The gut microbial composition was investigated by targeting the V3–V4 regions of the 16S rRNA gene for amplification and sequencing on the Illumina NovaSeq 6000 platform. A rarefaction curve was employed to assess sequencing depth, and the alpha diversity of each sample was quantified using the Chao1, Simpson, and Shannon indices based on the distribution of amplicon sequence variants (ASVs) across different samples. Additionally, beta diversity analysis was conducted to explore structural variations in microbial communities among different groups using unweighted UniFrac distance matrices. Principal coordinate analysis (PCoA) was employed for visualization, while permutational multivariate analysis of variance (PERMANOVA) was utilized to assess significant differences between groups. Furthermore, a taxonomic composition analysis was conducted to identify taxa with significantly different abundances among various groups.

### 2.17 Statistical Analysis

In this study, all statistical analyses were performed using GraphPad Prism 9.0. Normality tests were conducted on the data using the Shapiro–Wilk or Kolmogorov–Smirnov tests. Data were expressed as mean ± standard error of the mean (SEM) for normally distributed continuous variables. Comparisons among three or more independent groups were performed using the one‐way analysis of variance (ANOVA), followed by Dunnett's or Tukey's post hoc test. The data from different groups at different time points were compared using repeated measures two‐way ANOVA followed by Tukey's post hoc test. Non‐normally distributed variables were presented as median (interquartile range, IQR) and analyzed using the Kruskal‐Wallis test, followed by Dunn's post hoc test. Spearman correlation analysis was performed to examine the correlation between different outcomes. Statistical significance was considered at a *p*‐value less than 0.05.

## 3 Results

### 3.1 FMT Improved the Cognitive Function of 5 × FAD Mice

To evaluate the impact of FMT on AD‐like pathology, we initially conducted an experiment in which 5 × FAD mice received fecal microbiota capsules weekly for eight consecutive weeks (Figure S1A). Neither short‐term nor long‐term cognitive memory abilities showed significant improvements (Figure S1B–H). Furthermore, histological analyses revealed no significant reduction in hippocampal Aβ deposition, microglial activation, or colonic Aβ pathology (Figure S2A–G).

In response to these preliminary results, the experimental protocol was revised to administer FMT every other day over 8 weeks (Figure 1A). In this round of experiments, each mouse demonstrated a consistent decrease in the latency to escape and locate the platform zone during the training phase of the water maze test. Throughout the training stage, 5 × FAD mice displayed a remarkable delay in reaching the platform compared to their WT counterparts. Notably, compared to 5 × FAD mice, 5 × FAD‐FMT mice exhibited a shorter escape latency, indicating enhanced spatial learning and memory capabilities (Figure 1B). During the testing phase, 5 × FAD mice crossed the platform fewer times and explored the target quadrant for a shorter duration than WT mice. In contrast, 5 × FAD mice treated with FMT showed a significant increase in the frequency of platform crossings and the time spent exploring the target quadrant (Figure 1C,D), suggesting improved spatial memory function.

In the Y‐maze test, 5 × FAD mice demonstrated reduced exploration of the novel arm, as evidenced by fewer entries and shorter durations spent in this arm than WT mice. Conversely, 5 × FAD mice that underwent FMT exhibited enhanced exploration of the novel arm, characterized by an increased frequency of entries and prolonged durations of exploration (Figure 1E,F).

As for the novel object recognition test, 5 × FAD mice exhibited a significantly reduced discrimination index for novel objects compared to WT controls. However, a marked enhancement in the discrimination index was evident following the FMT intervention (Figure 1G,H). Overall, the results of the behavioral tests suggested that FMT ameliorated cognitive memory deficits, both short‐term and long‐term, in 5 × FAD mice.

### 3.2 FMT Altered the Composition of the Gut Microbiota in 5 × FAD Mice

**FIGURE 2:**
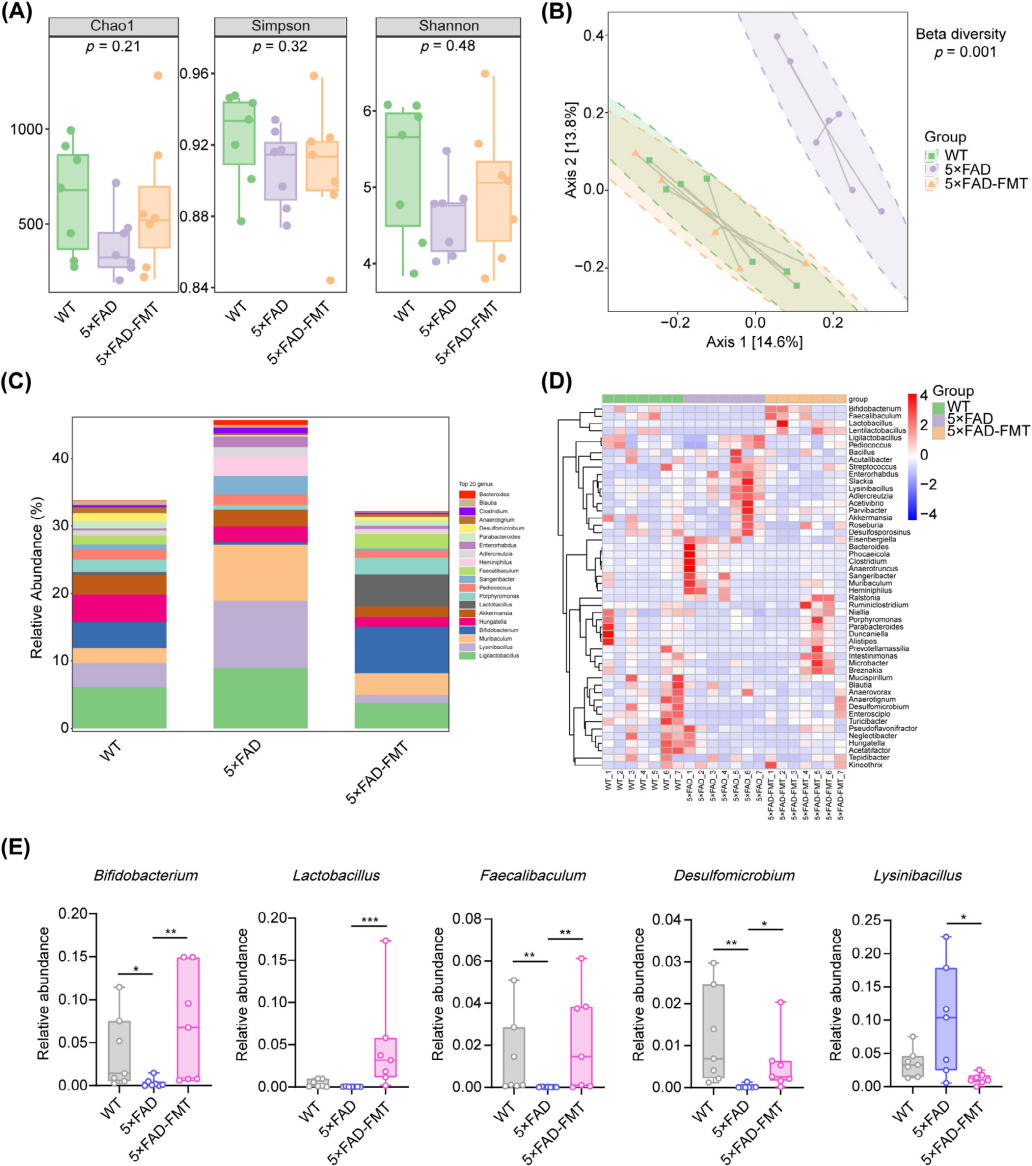
Alterations in gut microbiota composition in 5 × FAD mice following FMT. (A) Alpha diversity analysis of gut microbial richness (Chao1 index) and diversity (Simpson and Shannon indices) across different groups. (B) Beta diversity was assessed using PCoA at the ASV level to visualize intergroup differences in gut bacterial composition. PERMANOVA was employed to evaluate the statistical significance of these differences. (C) Taxonomic distribution of gut microbiota at the genus level. (D) Heatmap representation of the genus‐level composition of the gut microbiota, showing relative abundances across samples. (E) Quantitative analysis of the relative abundances of *Bifidobacterium*, *Lactobacillus*, *Faecalibaculum*, *Desulfomicrobium*, and *Lysinibacillus* among the three groups. *n* = 7 per group. Statistical analyses were conducted using the Kruskal‐Wallis test with Dunn's post hoc test for multiple comparisons. *p < 0.05, **p < 0.01, ***p < 0.001.

Previous research has identified alterations in gut microbial composition in AD mouse models [11, 36]. To confirm the presence of gut dysbiosis in 5 × FAD mice and to assess whether FMT could modulate the gut microbiota, 16S rRNA sequencing was employed. Alpha diversity analysis, encompassing the Chao1, Simpson, and Shannon indices, revealed no statistically significant variations in species richness or diversity among the three groups (Figure 2A). However, PCoA based on unweighted UniFrac distance matrices distinctly separated the microbial communities of 5 × FAD mice from those of WT controls. Notably, the gut microbial composition in 5 × FAD mice that underwent FMT closely matched that of the WT group, suggesting effective microbial integration (Figure 2B). A detailed taxonomic analysis was conducted to identify specific microbial genera exhibiting significant variations in abundance across the groups. The analysis showed that, compared to WT mice, the 5 × FAD group demonstrated a substantial decrease in the relative abundance of *Bifidobacterium*, *Faecalibaculum*, and *Desulfomicrobium*, accompanied by a concurrent increase in *Enterorhabdus*. Crucially, in 5 × FAD mice, FMT remarkably increased the relative abundance of *Bifidobacterium*, *Lactobacillus*, *Faecalibaculum*, and *Desulfomicrobium*, while concurrently decreasing *Lysinibacillus* (Figure 2C–E, Figure S3A–C). In conclusion, FMT induced alterations in the gut microbial composition in 5 × FAD mice.

### 3.3 FMT Reduced Inflammatory Responses and Aβ Pathology in the Colon of 5 × FAD Mice

**FIGURE 3:**
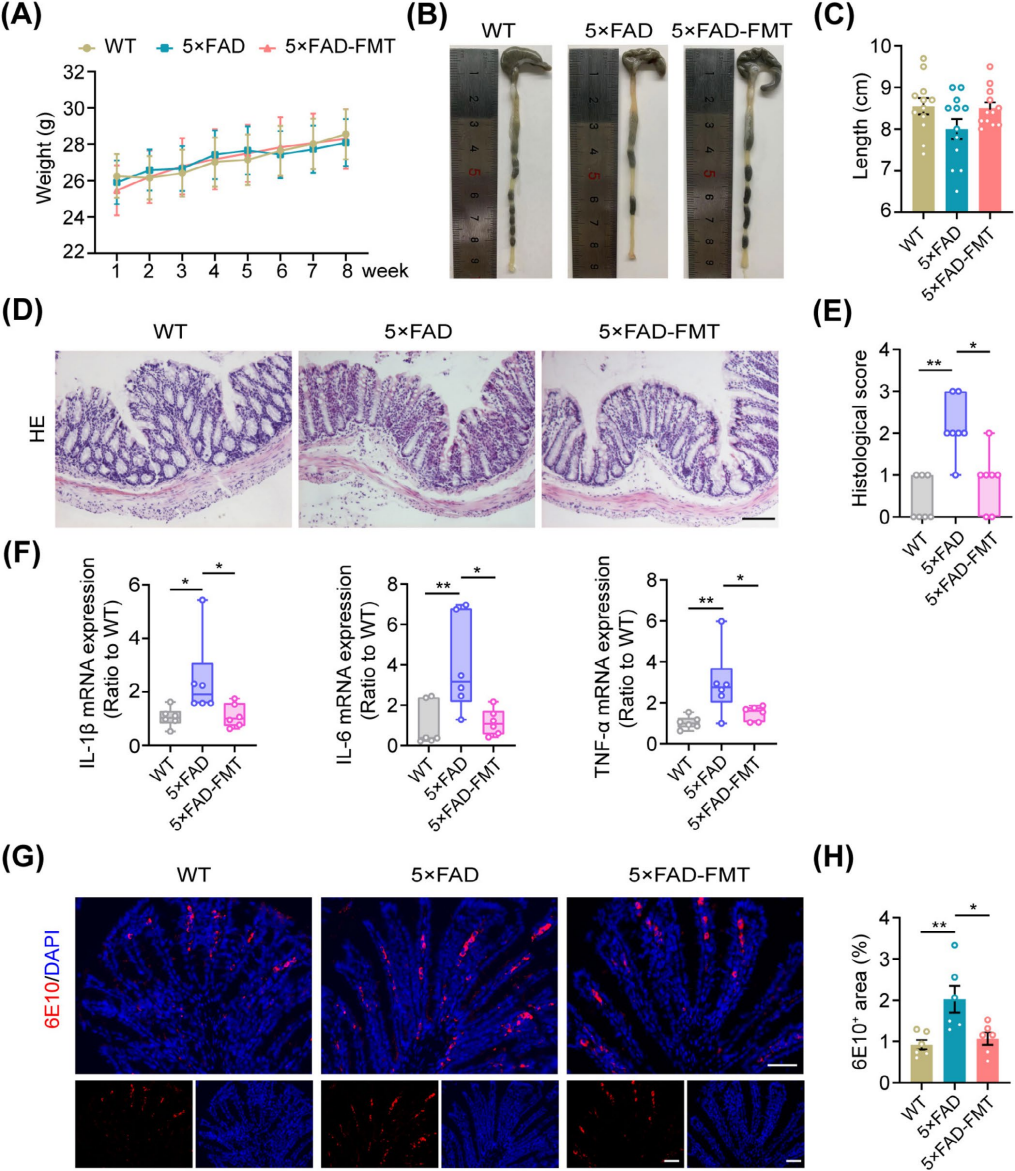
Ameliorative effects of FMT on colonic inflammatory responses and Aβ pathology in 5 × FAD mice. (A) Mean body weight changes in mice during the modeling phase. (B) Representative images of mouse colons. (C) Statistical analysis of colon length. (D) Representative images of HE staining in the colon. Scale bar, 100 μm. (E) Colonic histological scores based on HE staining. (F) Quantitative analysis of colonic IL‐1β, IL‐6, and TNF‐α mRNA expression levels. (G) Representative immunofluorescence images of 6E10 staining in the colon. Scale bar, 40 μm. (H) Percentage of 6E10^+^ area in the colon. *n* = 12 (A–C), *n* = 7 (E) and *n* = 6 (F–H) per group. Statistical significance was assessed using repeated measures two‐way ANOVA with Tukey's post hoc test for the weight data in (A), the Kruskal‐Wallis test with Dunn's post hoc analysis for the histological scores in (E) and relative mRNA expression levels of IL‐1β and IL‐6 in (F), and one‐way ANOVA with Dunnett's post hoc test for all other data. *p < 0.05, **p < 0.01.

During the modeling period, all mice within the three groups gained weight steadily (Figure 3A). We further conducted a comparative analysis of colon lengths among the three groups. 5 × FAD mice tended to have shorter colons than WT mice, although this difference did not reach statistical significance; nevertheless, after FMT, the difference was diminished (Figure 3B,C). HE staining revealed a greater level of colonic inflammatory infiltration in the 5 × FAD group compared to the WT group. However, FMT treatment effectively attenuated this inflammatory response (Figure 3D,E). Moreover, FMT significantly decreased the mRNA expression levels of IL‐1β, IL‐6, and TNF‐α in the colon of 5 × FAD mice, as demonstrated by qPCR analysis (Figure 3F).

Previous studies have revealed that intestinal epithelial cells can produce Aβ peptides [37, 38]. In line with these findings, our immunofluorescence and immunohistochemical analyses confirmed the presence of Aβ in colonic epithelial cells of mice. Moreover, 5 × FAD mice exhibited elevated levels of Aβ in colonic tissue compared to WT controls. Notably, following FMT treatment, a significant reduction in Aβ deposits was observed in the colon of 5 × FAD mice (Figure 3G,H, Figure S4A–C), suggesting the potential of FMT to alleviate colonic inflammation and Aβ pathology in these mice.

### 3.4 FMT Improved the Gut Barrier Integrity in 5 × FAD Mice

We next investigated the potential effects of changes in gut microbiota composition on gut barrier function. Given the critical role of tight junction proteins in maintaining gut barrier integrity, we used immunofluorescence labeling to identify zonula occluden‐1 (ZO‐1) and Claudin‐1 in the colon. The results demonstrated a remarkable upregulation of ZO‐1 and Claudin‐1 expression within the colon of 5 × FAD mice treated with FMT (Figure S5A–D). Moreover, FMT partially restored the expression levels of ZO‐1 and Occludin in the colon of 5 × FAD mice, as revealed by Western blot analysis (Figure S5E,F). These results suggest that FMT could potentially mitigate the disruption of gut barrier integrity in 5 × FAD mice by enhancing tight junction protein expression.

### 3.5 FMT Reduced Hippocampal Aβ Deposition and Neuroinflammation in 5 × FAD Mice

**FIGURE 4:**
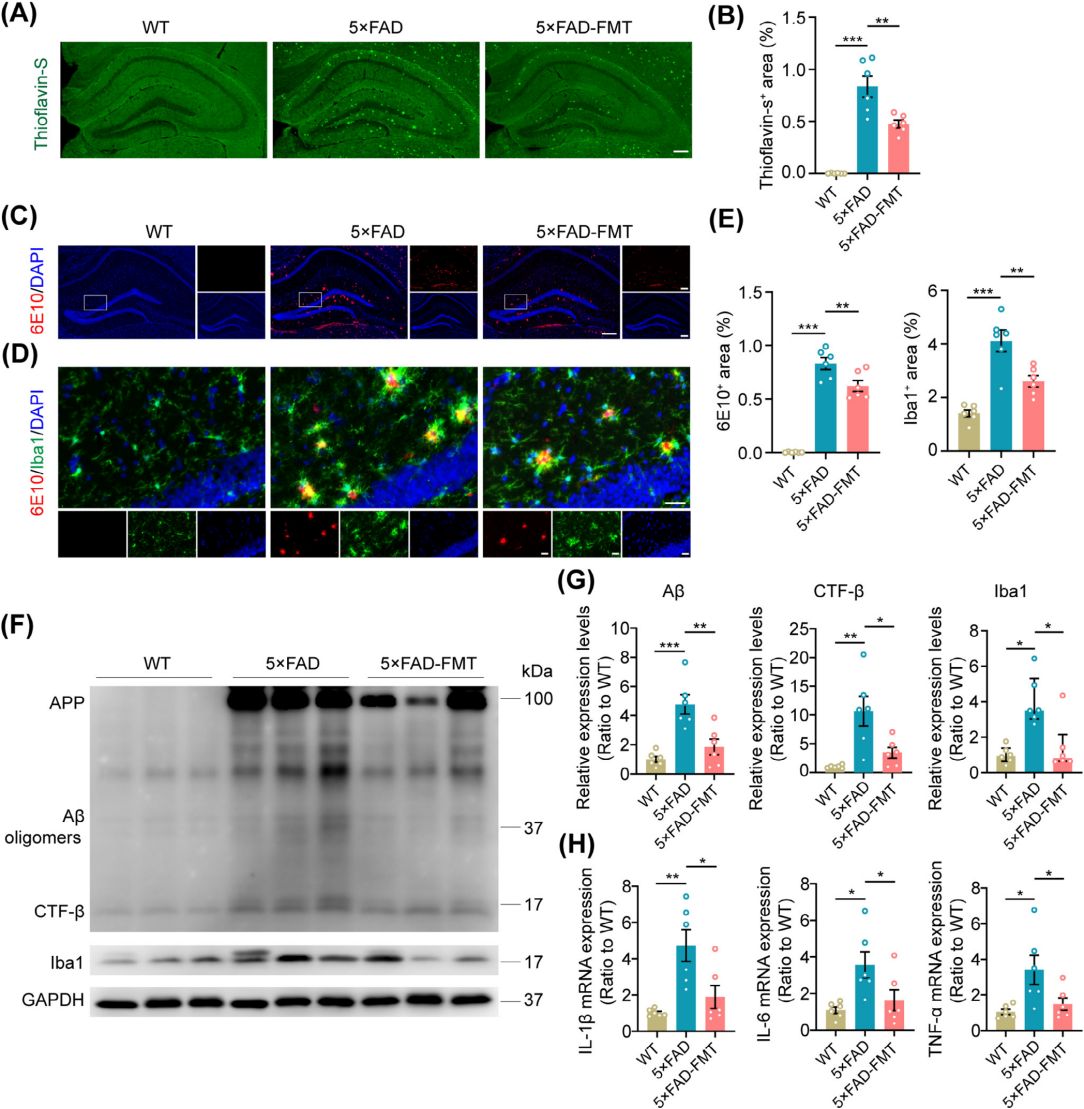
Improvements in hippocampal Aβ deposition and neuroinflammation following FMT in 5 × FAD mice. (A) Representative images showing Thioflavin‐S^+^ plaques in the hippocampus. Scale bar, 200 μm. (B) Quantitative analysis of the percentage of hippocampal Thioflavin‐S^+^ area. (C, D) Representative immunofluorescence images of the hippocampus stained with 6E10 (Red), Iba1 (Green), and DAPI (Blue) for each group. Scale bar, 200 μm for (C), and 40 μm for (D). (E) Statistical analysis of the percentage of hippocampal 6E10^+^ and Iba1^+^ area. (F, G) Representative Western blot bands and densitometry analysis of 6E10 and Iba1 in the hippocampus of all groups. (H) Statistical analysis of hippocampal IL‐1β, IL‐6, and TNF‐α mRNA expression levels. *n* = 6 per group. Statistical significance was evaluated using the Kruskal‐Wallis test with Dunn's post hoc analysis for Iba1 relative expression levels in (G) and one‐way ANOVA with Dunnett's post hoc test for all other data. *p < 0.05, **p < 0.01, ***p < 0.001.

To investigate how FMT affects brain pathology in 5 × FAD mouse models, Aβ deposition and microglial activation in the hippocampus were compared between untreated 5 × FAD mice and those receiving FMT. Following FMT, hippocampal Aβ plaque deposition was significantly reduced in 5 × FAD mice, as demonstrated by thioflavin‐S staining (Figure 4A,B). Immunofluorescence staining using the 6E10 antibody further supported this observation (Figure 4C–E). Immunofluorescence staining for Iba1 and microglial morphology analysis revealed that FMT treatment significantly reduced microglial activation in the hippocampus of 5 × FAD mice. This reduction was evidenced by a significant downregulation of Iba1 expression, an increase in the number of endpoints and total branches of microglia, and the elongation of these processes (Figure 4D,E, Figure S6A,B). Furthermore, 5 × FAD‐FMT mice exhibited lower levels of Aβ, β‐C‐terminal fragment (CTF‐β), and Iba1 expression in the hippocampal samples when compared with untreated 5 × FAD mice, as revealed by Western blot analysis (Figure 4F,G). qPCR analysis showed that FMT reduced the relative mRNA expression levels of IL‐1β, IL‐6, and TNF‐α in the hippocampal tissues of 5 × FAD transgenic mice (Figure 4H). These findings indicate that FMT effectively mitigated Aβ deposition and neuroinflammation in the hippocampus of 5 × FAD mice.

### 3.6 FMT Downregulated the Expression of Hippocampal APP, BACE1, and PS1 in 5 × FAD Mice

**FIGURE 5:**
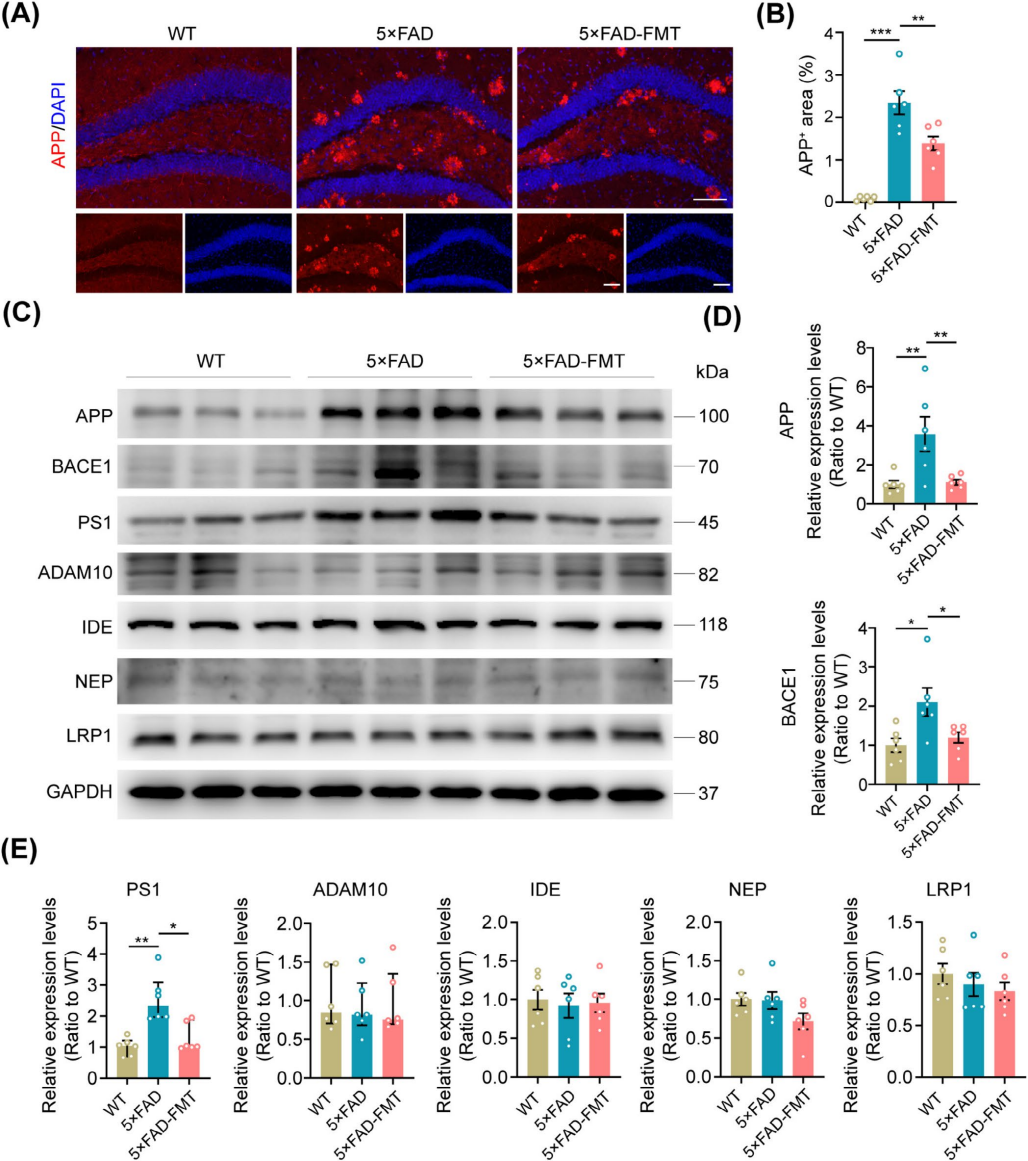
FMT‐induced APP, BACE1, and PS1 downregulation in the hippocampus of 5 × FAD mice. (A) Representative immunofluorescence images of APP in the hippocampus. Scale bar, 100 μm. (B) Statistical analysis of the percentage of hippocampal APP^+^ area. (C–E) Representative Western blot bands and densitometry analysis of APP, BACE1, PS1, ADAM10, IDE, NEP, and LRP1 in the hippocampus across the three groups. *n* = 6 per group. Statistical significance was evaluated using the Kruskal‐Wallis test with Dunn's post hoc analysis for relative expression levels of PS1 and ADAM10 shown in (E) and one‐way ANOVA with Dunnett's post hoc test for all other data. *p < 0.05, **p < 0.01, ***p < 0.001.

To further elucidate the precise mechanism by which FMT mitigates Aβ deposition within the hippocampus, immunofluorescence staining, and Western blot analysis were employed to quantify the levels of enzymes involved in Aβ metabolism. Immunofluorescence staining revealed a significant upregulation of APP expression in the hippocampus of 5 × FAD mice compared to WT controls. FMT intervention markedly reduced hippocampal APP levels in the 5 × FAD‐FMT group (Figure 5A,B). Western blot analysis corroborated these findings, demonstrating that FMT downregulated APP expression in the hippocampus of 5 × FAD mice. Moreover, both the primary β‐secretase, β‐site APP cleaving enzyme 1 (BACE1), and the catalytic subunit of γ‐secretase, presenilin‐1 (PS1), exhibited increased expression in the hippocampal tissues of 5 × FAD mice. Notably, FMT successfully decreased BACE1 and PS1 expression in the hippocampus of these mice, suggesting its potential role in attenuating amyloidogenic processing. No significant alterations were observed in the expression levels of a disintegrin and metalloprotease 10 (ADAM10), a key inhibitor of Aβ generation, insulin‐degrading enzyme (IDE), and neutral endopeptidase (NEP), both of which are involved in Aβ clearance, as well as low‐density lipoprotein receptor‐related protein 1 (LRP1) that facilitates Aβ transport (Figure 5C–E). These results indicate that FMT suppressed the expression of hippocampal APP, BACE1, and PS1 in 5 × FAD transgenic mice, thereby attenuating Aβ deposition.

### 3.7 FMT Inhibited the TLR4/IKKβ/NF‐κB Signaling Pathway in the Hippocampus of 5 × FAD Mice

**FIGURE 6:**
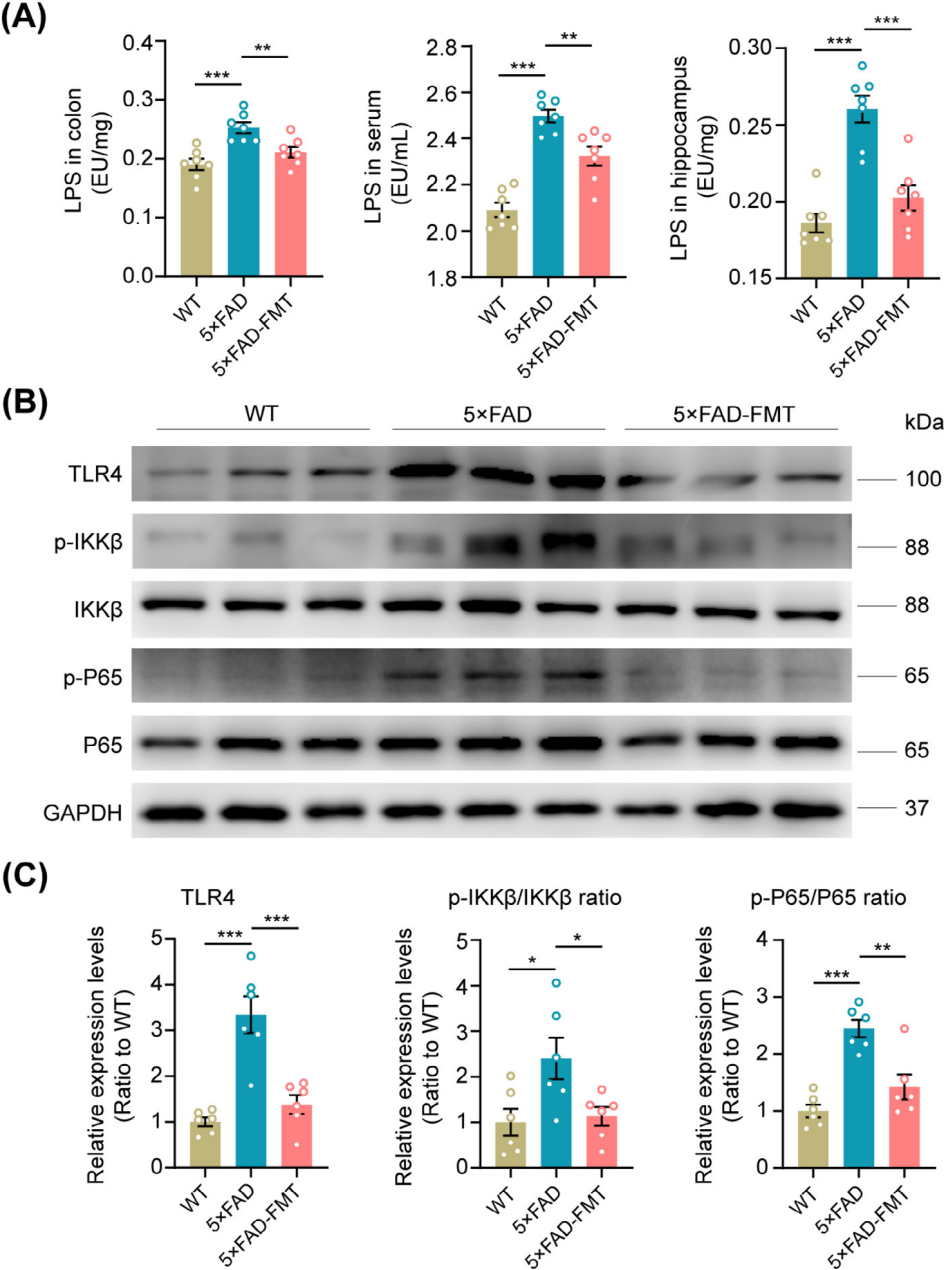
FMT‐induced suppression of the TLR4/IKKβ/NF‐κB signaling pathway in the hippocampus of 5 × FAD mice. (A) ELISA analysis of LPS levels in the colon, serum, and hippocampus. (B, C) Representative Western blot bands and densitometry analysis of hippocampal TLR4, p‐IKKβ/IKKβ, and p‐NF‐κB/NF‐κB. *n* = 7 (A) and *n* = 6 (B, C) per group. All statistical analyses were performed using one‐way ANOVA with Dunnett's post hoc test. *p < 0.05, **p < 0.01, ***p < 0.001.

To elucidate the mechanism underlying FMT's downregulation of APP, BACE1, and PS1 expression, we assessed the levels of LPS, a gut microbiome‐derived component, in the colon, serum, and hippocampus. LPS, originating from the gut microbiome, can translocate to the brain via a compromised gastrointestinal tract and BBB [39]. These molecules bind to microglial TLR4 upon entering the brain, triggering the downstream NF‐κB signaling pathway. This activation subsequently upregulates APP mRNA and enhances BACE1 activity, both crucial factors in Aβ production and AD pathology [18, 40]. ELISA results demonstrated that 5 × FAD mice exhibited significantly higher levels of LPS in the colon, serum, and hippocampal tissues compared to WT mice. Notably, FMT effectively suppressed these elevated LPS levels (Figure 6A). Western blot analysis revealed an activated state of the TLR4 pathway in the hippocampal tissues of 5 × FAD mice, characterized by significantly higher expression levels of TLR4, phosphorylated IKKβ (p‐IKKβ), and phosphorylated NF‐κB (p‐NF‐κB) compared to WT controls. However, FMT treatment in 5 × FAD mice significantly decreased the expression levels of these proteins (Figure 6B,C). In summary, the aforementioned evidence indicates that FMT effectively suppressed the activation of the hippocampal TLR4/IKKβ/NF‐κB signaling pathway mediated by LPS in 5 × FAD mouse models.

### 3.8 The Therapeutic Effect of FMT on Cognitive Impairment in 5 × FAD Mice Decreased With the Cessation of Treatment

To evaluate the sustained beneficial impact of FMT on cognitive decline in 5 × FAD mouse models, behavioral tests were conducted 4 weeks post‐FMT treatment (Figure S7A). No significant differences were observed in the latency to reach the platform between the 5 × FAD and 5 × FAD‐FMT groups throughout the training phase of the water maze test (Figure S7B). In contrast to 5 × FAD mouse models, 5 × FAD‐FMT mice showed an increased exploration time in the target quadrant during the testing period, while the number of platform crossings remained unchanged (Figure S7C,D). Additionally, 5 × FAD‐FMT mice displayed prolonged exploration of the novel arm in the Y‐maze test; however, there was no significant increase in the frequency of novel arm entries (Figure S7E,F). Similarly, in the novel object recognition test, no substantial differences were observed in the discrimination index toward the novel object between 5 × FAD‐FMT mice and their 5 × FAD counterparts (Figure S7G,H). Furthermore, the pathological analysis revealed that the long‐term protective effects of FMT on hippocampal Aβ deposition, microglial activation, and colonic Aβ pathology were not sustained (Figure S8A–G). These findings suggest that the mitigating effects of FMT on AD‐related memory impairment waned upon treatment termination.

### 3.9 Correlation Analysis Supported the Involvement of the Gut Microbiota‐LPS‐TLR4 Axis in the Cognitive Protective Effects of FMT on 5 × FAD Mice

**FIGURE 7:**
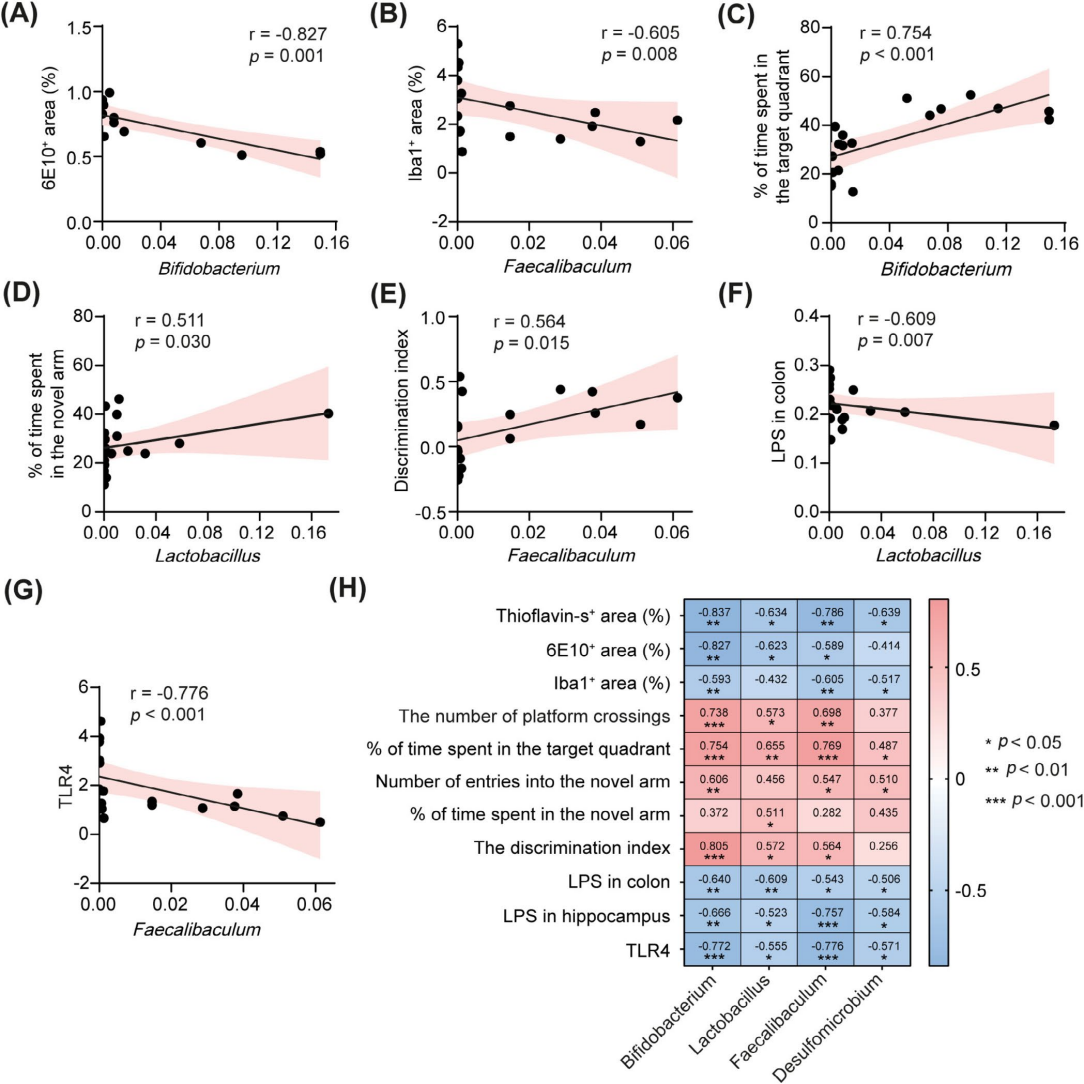
Correlation analyses between FMT‐altered gut microbiota relative abundance and various pathological and behavioral indicators. (A) The relative abundance of *Bifidobacterium* was negatively correlated with the percentage of 6E10^+^ area in the hippocampus. (B) The relative abundance of *Faecalibaculum* was negatively associated with the percentage of Iba1^+^ area in the hippocampus. (C) The relative abundance of *Bifidobacterium* was positively correlated with the percentage of time spent in the target quadrant in the Morris water maze test. (D) The relative abundance of *Lactobacillus* was positively correlated with the percentage of time spent exploring the novel arm in the Y‐maze test. (E) The relative abundance of *Faecalibaculum* was positively correlated with the discrimination index in the novel object recognition test. (F) The relative abundance of *Lactobacillus* was inversely correlated with LPS levels in the colon. (G) The relative abundance of *Faecalibaculum* was negatively correlated with TLR4 expression levels in the hippocampus. (H) Heatmap of correlation analyses. All analyses were performed using Spearman correlation analysis. *p < 0.05, **p < 0.01, ***p < 0.001.

We further conducted a correlation analysis to investigate the relationship between FMT‐induced alterations in gut microbiota and various experimental outcomes. Our results revealed a significant negative correlation between the relative abundances of specific microbiota genera, including *Bifidobacterium*, *Lactobacillus*, *Faecalibaculum*, and *Desulfomicrobium*, and both Aβ deposition and microglial activation in the hippocampus (Figure 7A,B,H). Furthermore, the relative abundances of these bacterial taxa positively correlated with cognitive performance, as assessed by the Morris water maze, Y‐maze, and novel object recognition test (Figure 7C–E,H), suggesting their potential role in cognitive enhancement. Additionally, we identified a significant negative correlation between colonic LPS levels and the relative abundances of *Bifidobacterium* and *Lactobacillus* (Figure 7F,H). A similar negative correlation was observed between hippocampal LPS levels and the relative abundances of *Bifidobacterium* and *Faecalibaculum* (Figure 7H). Remarkably, there was also a negative correlation between the expression levels of TLR4 in the hippocampus and the relative abundances of *Bifidobacterium* and *Faecalibaculum* (Figure 7G,H). These findings collectively support the hypothesis that FMT may protect against cognitive deficits in 5 × FAD mice by modulating the gut microbiota‐LPS‐TLR4 axis.

### 3.10 The Supplementation of LPS Counteracted the Positive Impacts of FMT on Aβ Pathology and Microglial Activation in the Hippocampus of 5 × FAD Mice

**FIGURE 8:**
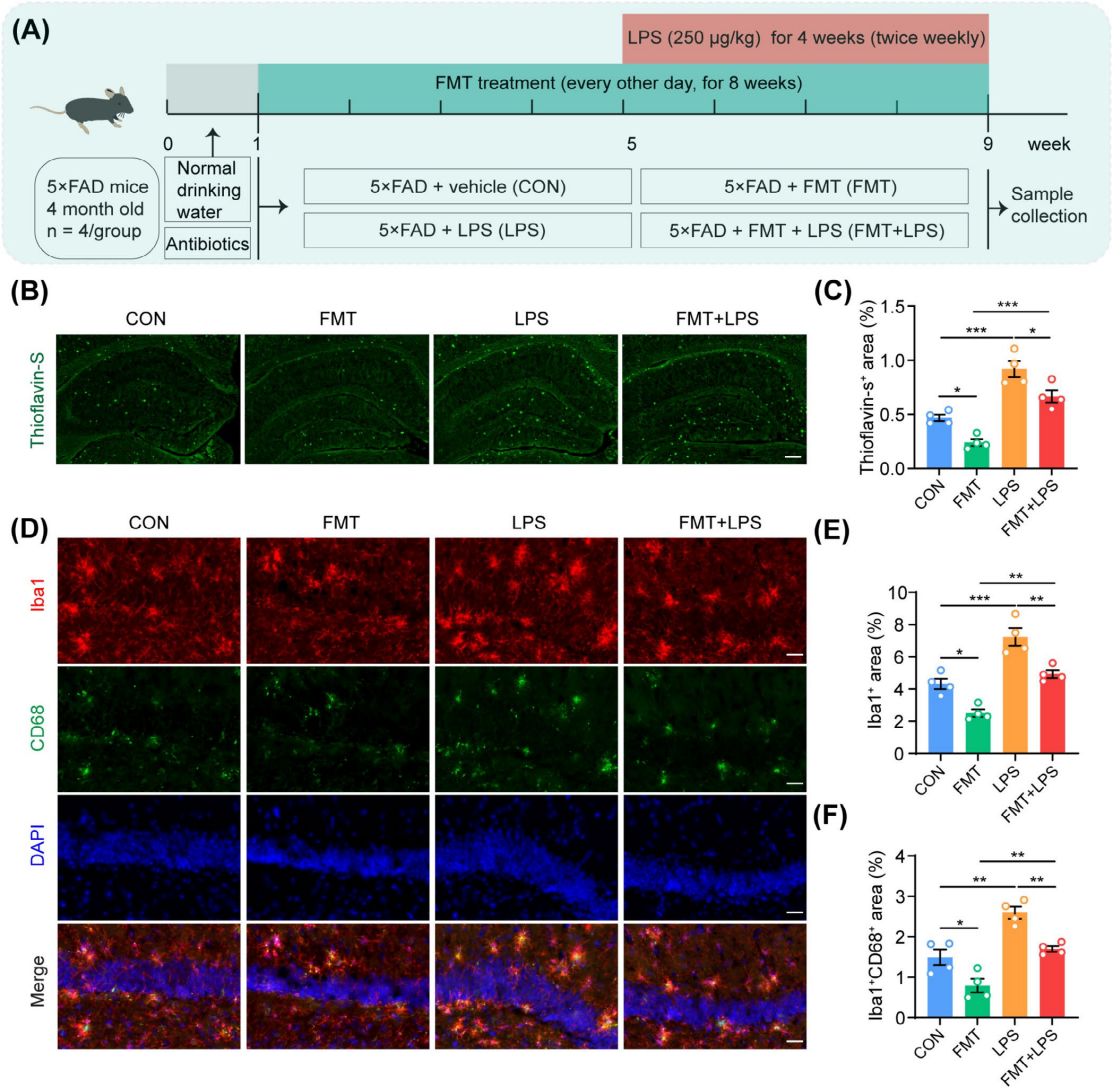
LPS supplementation attenuated the beneficial effects of FMT on AD‐like pathology in 5 × FAD mice. (A) Flowchart of the supplementary LPS experiment. (B) Representative images of Thioflavin‐S staining in the hippocampus. Scale bar, 200 μm. (C) Statistical analysis of the percentage of Thioflavin‐S^+^ area in the hippocampus. (D) Representative immunofluorescence images of Iba1 (Red), CD68 (Green), and DAPI (Blue) in the hippocampus of each group. Scale bar, 40 μm. (E) Statistical analysis of the percentage of Iba1^+^ area. (F) Statistical analysis of the percentage of Iba1^+^CD68^+^ area in the hippocampus. *n* = 4 per group. Statistical significance was evaluated using one‐way ANOVA with Tukey's post hoc test. *p < 0.05, **p < 0.01, ***p < 0.001.

To verify that FMT alleviates AD‐like pathology by reducing LPS levels, 5 × FAD mice underwent FMT and were subsequently administered LPS intraperitoneally (Figure 8A). The results indicated that FMT significantly reduced thioflavin‐S‐positive Aβ plaques in the hippocampus of these mice (Figure 8B,C). Conversely, LPS intervention alone notably exacerbated Aβ deposition in the hippocampus (Figure 8B,C). Interestingly, supplementing with LPS following FMT treatment substantially counteracted the therapeutic benefits of FMT in reducing hippocampal Aβ pathology in 5 × FAD mice (Figure 8B,C). Furthermore, immunofluorescence staining for Iba1 and CD68 demonstrated that intraperitoneal administration of LPS reversed the ameliorative effects of FMT on microglial activation in the hippocampus of 5 × FAD mice, as evidenced by an increase in Iba1‐positive microglia and colocalization of Iba1 with CD68 (Figure 8D–F). In summary, LPS supplementation significantly attenuated the beneficial effects of FMT on AD‐like pathology, further corroborating the mechanism by which FMT mitigates AD pathology by reducing LPS levels in the gut and hippocampus.

## 4 Discussion

The etiology of AD is highly complex, with a lack of effective treatments currently available [41, 42]. This situation underscores the urgent need to explore promising therapeutic strategies for AD. It is generally accepted that abnormal Aβ generation and aggregation in the brain is the primary cause of AD [43]. Aβ peptides are produced by sequential cleavage of APP by β‐ and γ‐secretase [17, 44, 45]. In this work, we employed 5 × FAD transgenic mice, an early‐onset model of amyloid pathology in which intraneuronal Aβ deposition initiates at the age of 1.5 months, preceding memory deterioration at 4–5 months [26].

Previous studies have demonstrated significant differences in gut microbial composition between transgenic mouse models of AD and their WT counterparts and between individuals with AD and their healthy controls [8, 9, 11, 36]. Notably, the relative abundances of *Firmicutes*, *Bifidobacteria*, and *Lactobacillus* are reduced in 5 × FAD mice, while the abundance of *Bacteroidetes* is increased [42]. Similarly, the microbiome of AD patients exhibits a reduction in the abundance of *Firmicutes* and *Bifidobacterium*, accompanied by an increase in *Bacteroidetes* compared to non‐AD controls [8]. Consistently, our results demonstrated a significant decrease in the abundance of *Bifidobacterium*, *Faecalibaculum*, and *Desulfomicrobium* in 5 × FAD mice compared to WT mice. Furthermore, in APP/PS1 mice, colonization with fecal bacteria from WT mice has been shown to reverse gut microbiota composition, mitigate AD pathology, and improve cognitive impairment [24]. Similarly, ADLP^APT^ mice, which exhibit abnormal Aβ deposition and neurofibrillary tangle formation in the brain, have improved pathological and cognitive deficits following FMT from WT mice [23]. These findings collectively suggest that restoring a healthy gut microbiota may alleviate the pathological processes associated with AD. Consistent with these findings, the present results demonstrate a dramatic attenuation of cognitive deficits, a core symptom of AD, in 5 × FAD mice after transplantation of a healthy human gut microbiota.

Current studies reveal inconsistent patterns of gut microbiota alterations in AD mouse models following FMT. Notably, when cecal suspensions from young and aged WT mice were transplanted into 5 × FAD mice, significant changes in the abundance of *Firmicutes*, *Lactobacillaceae*, and *Bifidobacteria* were observed in the recipient animals [46]. Conversely, FMT from 5 × FAD mice to normal C57BL/6 mice led to decreased levels of *Burkholderiales*, *Barnesiella*, and *Prevotella* while increasing the abundance of *Rikenella* [47]. APP/PS1 mice receiving FMT from WT mice demonstrated decreased abundances of *Proteobacteria* and *Verrucomicrobia* but increased levels of *Bacteroidetes* [24]. Our study showed that FMT significantly increased the relative abundance of *Bifidobacterium*, *Lactobacillus, Faecalibaculum*, and *Desulfomicrobium* in 5 × FAD transgenic mice, aligning with some of the abovementioned findings. *Bifidobacterium* and *Lactobacillus* are widely used as probiotics to prevent or treat specific diseases. Independent studies reported that the combined administration of *Bifidobacterium* and *Lactobacillus* significantly alleviated pathological impairments and cognitive deficits in aged senescence‐accelerated mouse‐prone 8 (SAMP8) mice [48, 49]. The probiotic blend SLAB51, containing *Bifidobacterium* and *Lactobacillus*, has been shown to modulate the composition of gut microbiota and their metabolites in 3 × Tg‐AD mice, thereby impeding neurodegenerative progression [50]. Furthermore, it has been observed that these two probiotics can ameliorate cognitive impairment in 5 × FAD mice by inhibiting LPS‐producing gut microbiota [51]. Several clinical studies have also underscored the therapeutic potential of these probiotics [52, 53, 54]. For instance, a multicenter trial on individuals aged over 65 years revealed that *Bifidobacterium* could potentially maintain psychological and cognitive health by modulating gut microbiota [52]. *Faecalibaculum*, an obligate anaerobe, plays a significant role in lactate production in middle‐aged mice, with a potential inverse relationship between intestinal lactate dominance and aging [55]. Additionally, it has been reported that *Faecalibaculum* secretes short‐chain fatty acids, which have been proven effective in preventing and improving intestinal inflammation [56, 57, 58]. These findings suggest that *Bifidobacterium*, *Faecalibaculum*, and *Lactobacillus* exhibit promising prospects for alleviating intestinal inflammation and cognitive decline associated with AD. Moreover, hydrogen sulfide, a metabolic end product of *Desulfomicrobium* [59], is a crucial neuromodulator that positively impacts mitochondrial bioenergetics at physiological concentrations [60]. A reduction in hydrogen sulfide levels has been observed in brain tissue from AD patients [61] and mouse models [62], while supplementing with hydrogen sulfide exhibits neuroprotective effects in an AD model [60]. Notably, hydrogen sulfide decreases the expression of the amyloidogenic C99 fragment, BACE1, and PS1 in APP/PS1 mice [63]. Therefore, we speculate that the increased abundance of *Desulfomicrobium* following FMT in our study may potentially play a role in suppressing Aβ‐producing enzymes, ultimately ameliorating AD‐like pathology in 5 × FAD mice.

A recent review summarizes that microglia and gut microbiota play a dual‐edged role in AD [64]. On the one hand, AD patients suffer from intestinal dysbiosis, characterized by decreased beneficial microbial communities and increased potentially pathogenic ones [8, 9, 65], impairing both the intestinal epithelial barrier and BBB [66, 67]. Early in the disease, AD patients exhibit damage to the BBB [68]. Amyloid proteins and LPS produced by gut microbiota may penetrate the bloodstream via the damaged intestinal barrier and cross the BBB into the brain. This process activates peripheral immune cells and microglia, triggering systemic and neuroinflammation, thereby exacerbating AD pathological damage [18, 69]. On the other hand, gut microbiota‐targeting therapies, like FMT, probiotics, and prebiotics, have demonstrated therapeutic potential in mitigating neuroinflammation and halting AD progression [6, 70]. ADLP^APT^ mice that received FMT from WT mice showed a significant reduction in the activation of microglia and astrocytes, a decrease in Aβ plaques, and improved spatial learning and memory function [23]. The administration of *Agathobaculum butyriciproducens* (SR79), a butyric acid‐producing bacterium, has been found to ameliorate microglial activation and cognitive impairments in APP/PS1 transgenic and LPS‐induced cognitive impairment mouse models [71]. In this study, we observed that FMT effectively altered the gut microbiota composition of 5 × FAD mice, reversing their increased intestinal permeability. Notably, following FMT, hippocampal LPS levels decreased, potentially reducing the number of pro‐inflammatory microglia or facilitating their conversion to an anti‐inflammatory phenotype. Thus, understanding the interactions between gut microbiota, microglia, and neuroinflammation may help guide AD treatment.

Currently, the primary therapeutic mechanisms of FMT in AD revolve around its regulatory effects on microbial metabolites [24, 72], immune responses [23], and both colonic and neuroinflammatory processes [23, 47, 73]. Previous research in a mouse model of Parkinson's disease has demonstrated that FMT can effectively reduce intestinal pathogenic LPS levels and, via the gut‐brain axis, diminish LPS concentrations in the substantia nigra [29]. However, whether FMT can achieve similar outcomes in the 5 × FAD mouse model remains unknown. Our study is the first to demonstrate that FMT‐induced alterations in the gut microbiota composition of 5 × FAD mice result in reduced LPS levels in the colon, blood circulation, and hippocampus, ultimately inhibiting the TLR4‐NF‐κB signaling pathway. Furthermore, given the limited research on FMT's regulatory mechanisms concerning Aβ metabolism, we focused on the modulatory role of NF‐κB on Aβ‐metabolizing enzymes. This investigation provides evidence of FMT's beneficial impact on Aβ pathology by downregulating enzymes involved in Aβ production.

LPS, a crucial component of the Gram‐negative bacterial cell wall, can trigger inflammatory responses of varying severity when released into the bloodstream [74]. Research in C57BL/6J mice has shown that intraperitoneal LPS injection induces systemic and neuroinflammatory responses, leading to Aβ formation, neuronal loss, and cognitive impairment [75]. Clinical studies have also revealed elevated serum and brain LPS levels in AD patients compared to healthy controls [76, 77, 78]. Additionally, LPS colocalizes with Aβ_1‐40/42_ and amyloid plaques in Alzheimer's brains, suggesting a potential link between AD pathology and this Gram‐negative bacterial molecule [40, 76]. Prolonged exposure to bacterial LPS in mice has been shown to induce AD‐like pathology [79]. Interestingly, research has shown that *Bifidobacterium longum and Lactobacillus plantarum* significantly inhibit the production of LPS by *Escherichia coli* K1 in vitro. Mice exposed to *E. coli* K1 or LPS develop intestinal dysbiosis and cognitive decline, which can be alleviated by administering these probiotic strains [80]. These findings suggest a potential association between LPS and AD progression, with probiotics offering a promising strategy to mitigate AD advancement by suppressing LPS production. Our data aligned with these previous studies, demonstrating elevated LPS levels in the serum, colon, and hippocampus of 5 × FAD mice compared to WT controls. FMT treatment significantly reduced LPS levels in these tissues of 5 × FAD mice. The notable increase in *Bifidobacterium*, *Faecalibaculum*, and *Lactobacillus* following FMT in our study may contribute to inhibiting LPS production by intestinal Gram‐negative bacteria in 5 × FAD mice, potentially offering therapeutic benefits for AD‐like pathology.

LPS, a classical pathogen‐associated molecular pattern (PAMP), binds to microglial surface pattern recognition receptors (PRRs), mainly TLR4, triggering activation of the NF‐κB pathway and subsequent release of TNF‐α, IL‐1β, and IL‐6. These cascades can upregulate APP expression and BACE1 activity, thereby augmenting Aβ production [18, 81]. Consequently, we focused on examining the impact of FMT on the hippocampal TLR4/IKKβ/NF‐κB signaling pathway to gain insight into the underlying mechanisms of its neuroprotective effects against cognitive deficit in 5 × FAD mice. Notably, FMT treatment effectively suppressed NF‐κB pathway activation in the hippocampus. Additionally, FMT decreased APP, BACE1, and PS1 expression levels, reducing Aβ production. However, FMT did not impact Aβ clearance enzymes. As a result, this intervention alleviated Aβ accumulation and deposition. Previous research suggests that NF‐κB may upregulate BACE1 promoter activity, increasing BACE1 transcription [45, 82]. Furthermore, under conditions of supraphysiological Aβ concentrations, NF‐κB activation has been shown to promote the transcription of APP, PS1, and BACE1 and even enhance their expression and enzymatic activity. While it remains uncertain whether NF‐κB directly regulates APP and PS1 transcription [83, 84, 85], inhibiting NF‐κB activity can promote the breakdown of APP and BACE1 through proteasomal and lysosomal pathways [86]. These results indicate a complex interplay among NF‐κB, inflammatory cytokines, and the modulation of critical proteins involved in AD pathogenesis.

Aβ has been detected in mouse intestinal epithelial cells [37, 38], and overnight LPS stimulation can enhance the basal secretion of Aβ_1–42_ and Aβ_1–40_ in human colon epithelial cells in vitro [87]. Intestinal inflammation can induce local Aβ production within the gut [88]. In this study, immune‐positive signals for Aβ were consistently observed in the colon epithelial cells of 6‐month‐old WT mice; however, these signals were more pronounced in age‐matched 5 × FAD mice. FMT has been observed to attenuate the colonic Aβ burden of 5 × FAD mice. While the precise mechanism remains unclear, it is reasonable to speculate that the increased probiotics, reduced LPS levels, and improved intestinal inflammatory environment mediated by FMT could be critical factors in inhibiting colonic Aβ secretion. Given the potential of the vagus nerve and blood to transport gut‐derived Aβ to the brain [88, 89], reduced intestinal Aβ burden may potentially contribute to mitigating AD pathology.

Importantly, every‐other‐day FMT intervention in the current 8‐week schedule significantly improved AD pathology and symptoms. In contrast, weekly administrations failed to produce comparable improvements, hinting at the dose‐dependent effectiveness of FMT therapy. Given the complex interplay between donor and recipient microbiota in FMT, recent studies indicate that increasing the number of donor microorganisms could enhance colonization in the recipient's intestines [90]. This strategy is supported by research showing that high‐dose or repeated FMT improves colonization efficiency and potency [91, 92, 93]. The capsule‐based FMT approach in this research offers a significant advantage over invasive techniques like gastrointestinal tubes and colonoscopy, enabling more frequent FMT administrations [94]. Currently, data on the long‐term effectiveness of FMT therapy specifically for AD remain scarce. This study, for the first time, elucidates the dose‐effectiveness and time‐effectiveness characteristics of FMT in treating AD‐like pathology. Smillie et al. identified 125 donor‐derived strains at the initial follow‐up post‐FMT. Still, this number decreased to 58 strains beyond 1 month, suggesting a potential decline in FMT efficacy over time [95]. While moderate to severe irritable bowel syndrome symptoms may improve within 3 months after FMT intervention, the effect is significantly reduced after 12 months [96]. FMT exhibits pronounced short‐term benefits for metabolic syndrome and chronic gastrointestinal diseases, but its long‐term clinical impact remains limited [97, 98]. Nevertheless, previous research has shown that a single FMT can improve clinical outcomes in AD and Parkinson's disease, with these improvements lasting up to 6 months following treatment [99, 100]. Despite this, there is still a lack of agreement on standardized procedures for FMT implementation, and the number of transplants administered in each case varies widely from one to tens of rounds [7]. Recently, it has been reported that, unlike *Clostridium difficile* infection, gut dysbiosis represents only one of several pathogenic factors in chronic non‐infectious diseases. FMT efficacy in these conditions is usually not long‐lasting, necessitating multiple rounds of treatment [90, 101]. In addition to the dosage and frequency of transplantation, the composition of donor microorganisms, the microbial compatibility between donor and recipient, and the high abundance of specific strains all serve as critical determinants of FMT efficacy [90]. This study suggests that the protective effects of FMT on cognitive function in 5 × FAD mice are not sustained beyond 4 weeks post‐FMT cessation, possibly due to the limited microbial similarity between human donors and mouse recipients.

It should be noted that a substantial body of preclinical studies have consistently demonstrated the cognitive‐protective effects of FMT and probiotic treatments in AD mouse models [23, 24, 25, 48, 49, 50, 51]. Probiotics, particularly *Bifidobacterium* and *Lactobacillus*, have exhibited promising therapeutic potential in alleviating cognitive impairments associated with AD [53, 54]. However, clinical exploration of FMT as a treatment for AD remains extremely limited, with only a few case reports available and no published clinical trials to date [102]. Differences in gut microbiota between rodents and humans, along with the challenges in selecting appropriate transplant donors, pose significant obstacles to the clinical translation research of FMT. Consequently, the application of FMT in the clinical practice of AD remains a distant goal, necessitating large‐scale clinical trials to assess the efficacy and safety of FMT for AD treatment.

Based on this, the present study has several limitations. First, interspecies differences in gut microbiota composition may hinder the colonization efficacy of donor strains in FMT. Second, FMT's effects may involve modulating gut microbial metabolite synthesis, an aspect not assessed in this trial. Finally, the study did not examine the changes in gut microbiota 4 weeks post‐FMT. Further research is needed to comprehensively understand FMT's mechanisms and factors influencing its long‐term efficacy.

In conclusion, FMT successfully alleviates cognitive impairments in 5 × FAD mice by modulating their gut microbiota composition. Our findings highlight dose‐ and time‐dependent characteristics of FMT in treating AD. Mechanistic studies suggest that the protective effect of FMT on AD may primarily be attributed to its inhibition of the LPS‐TLR4 pathway, which subsequently ameliorates both intestinal and neuroinflammation and suppresses Aβ production. This study provides a novel perspective for exploring potential preventive and therapeutic approaches for AD.

## Author Contributions

Ting Wu, Yu Zheng, Yini Dang, and Ming Xiao conceived and designed the experiments. Xueqin Jiang, Yu Zheng, Huaiqing Sun, and Mengmei Yin conducted pathological experiments and behavior tests. Ming Xiao and Xueqin Jiang wrote and modified the manuscript. All the authors contributed to the review of the manuscript.

## Conflicts of Interest

The authors declare no conflicts of interest.

## Supporting information


Appendix S1.

## Data Availability

The data that support the findings of this study are available from the corresponding author upon reasonable request.
