# Supplementary material for: Fecal Microbiota Transplantation Improves Cognitive Function of a Mouse Model of Alzheimer's Disease
Source: CNS Neurosci Ther. 2025 Feb 17;31(2):e70259. doi: 10.1111/cns.70259 (PMC11831070; doi:10.1111/cns.70259)
Supplement: Supplementary file 1 — Appendix S1. [file CNS-31-e70259-s001.zip › cns70259-sup-0002-DataS1.pdf]

Figure S5E

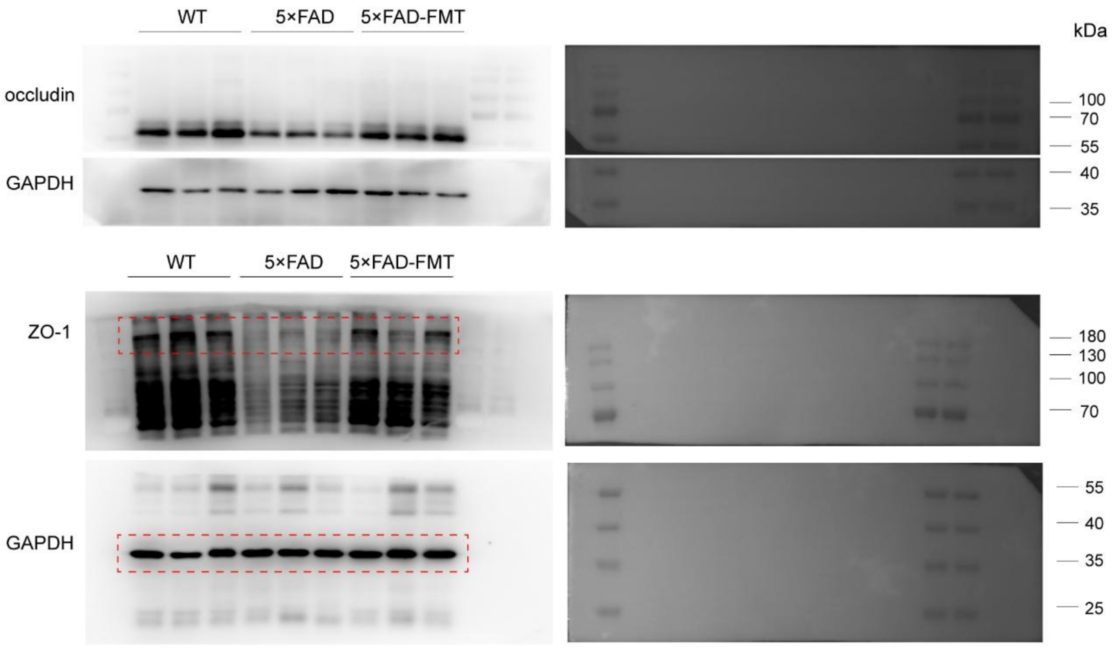

Full unedited blots for Figure S5E

Figure 4F

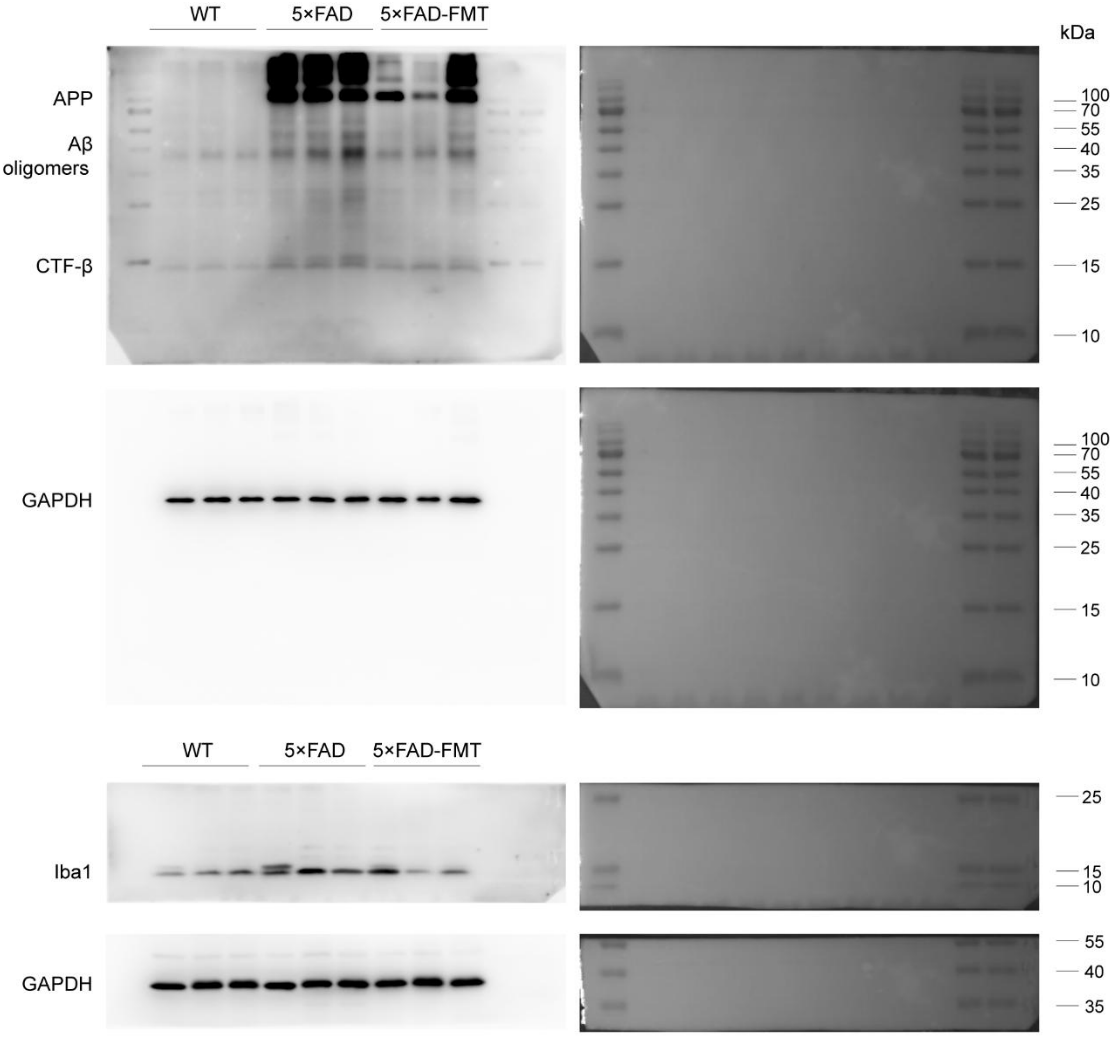

Full unedited blots for Figure 4F

Figure 5C

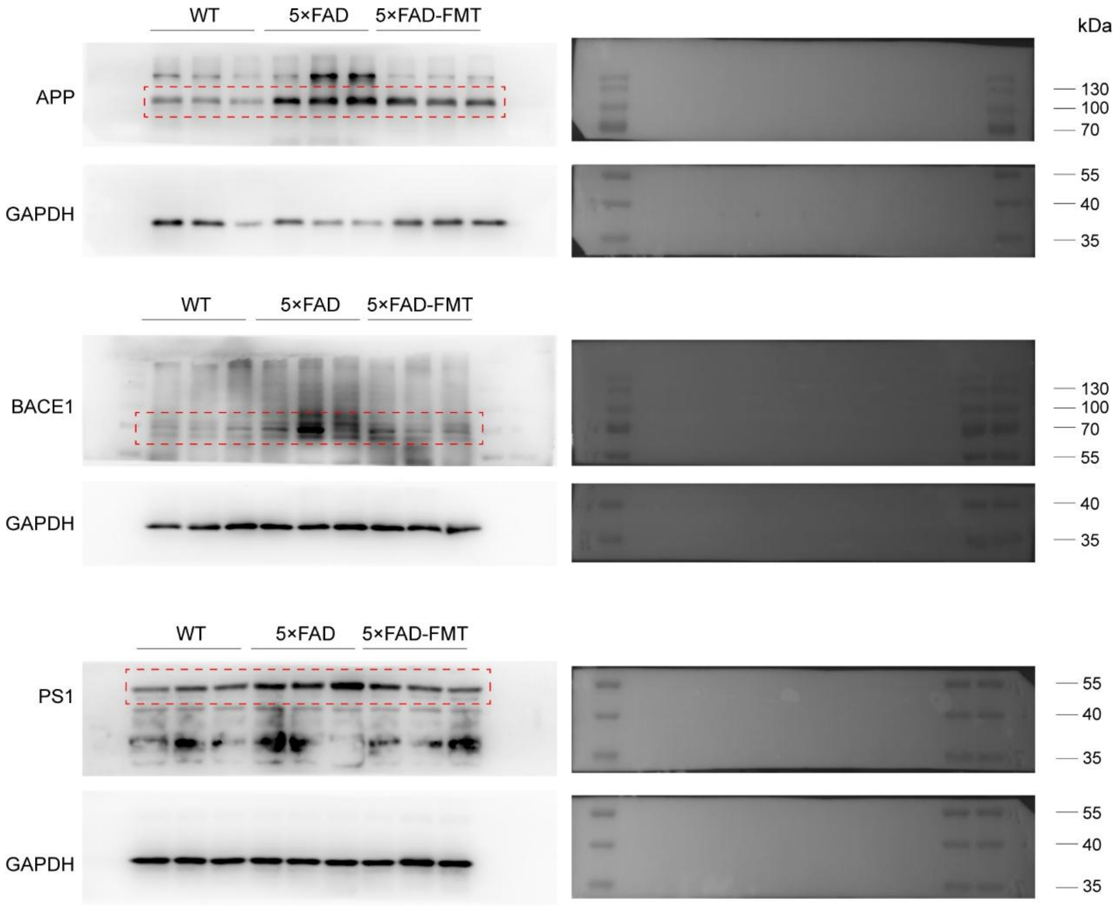

Full unedited blots for Figure 5C

Figure 5C

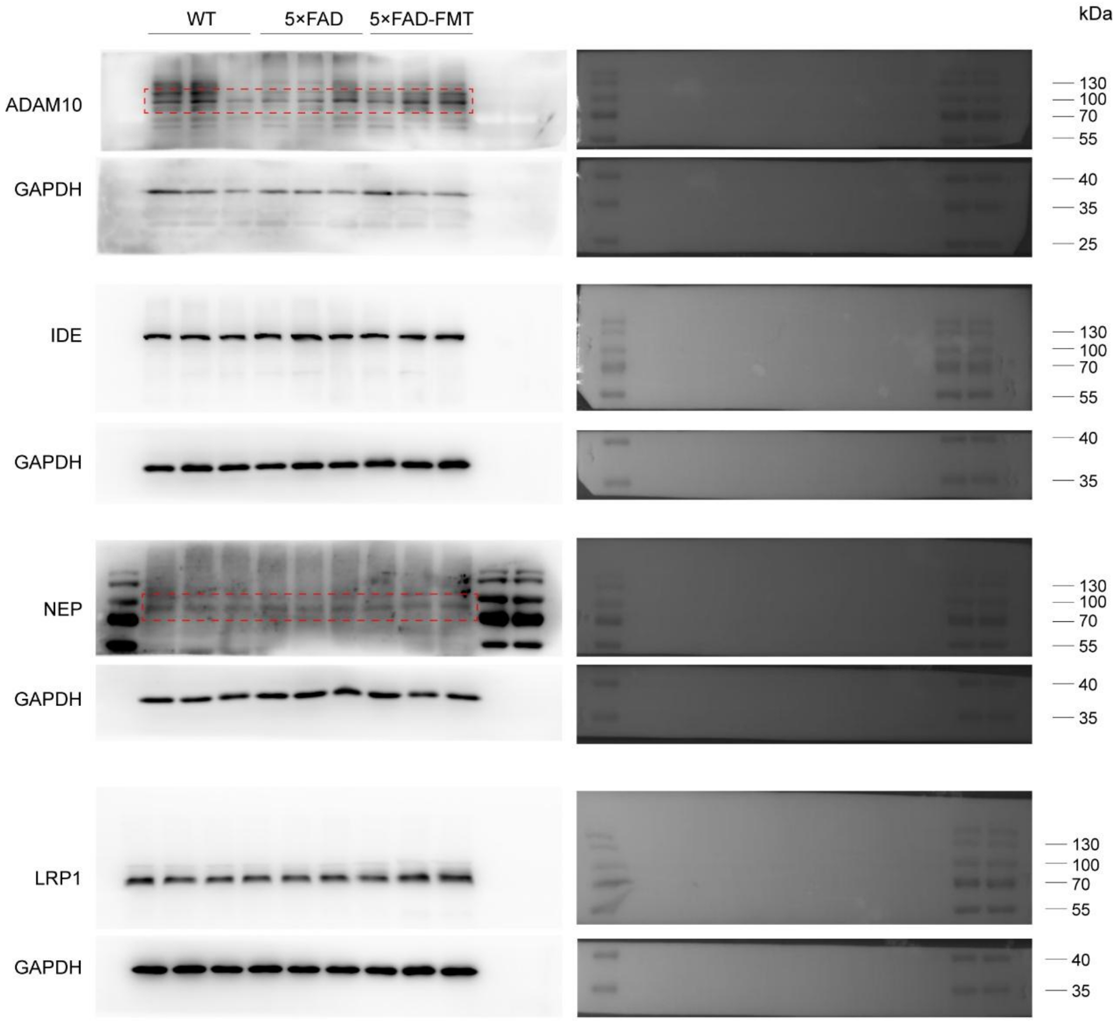

Full unedited blots for Figure 5C

Figure 6B

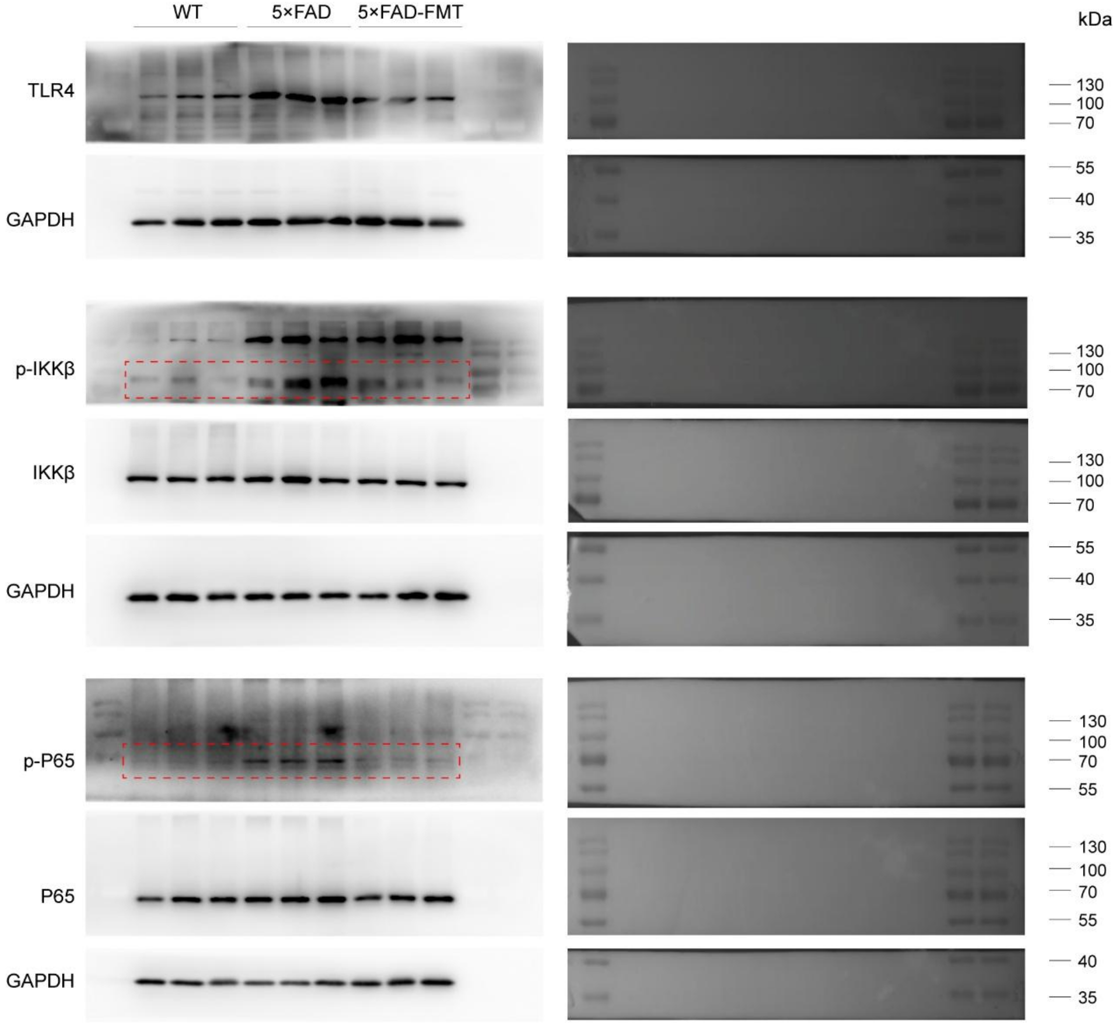

Full unedited blots for Figure 6B
