# Supplementary material for: Fecal Microbiota Transplantation Improves Cognitive Function of a Mouse Model of Alzheimer's Disease
Source: CNS Neurosci Ther. 2025 Feb 17;31(2):e70259. doi: 10.1111/cns.70259 (PMC11831070; doi:10.1111/cns.70259)
Supplement: Supplementary file 1 — Appendix S1. [file CNS-31-e70259-s001.zip › cns70259-sup-0001-AppendixS1.docx]

**Table S1: Information on the fecal microbiota capsule**

| Bacterial type | Genus | Sequence number | Coverage (%) |
| --- | --- | --- | --- |
| G^+^ | *Bifidobacterium* | 836 | 93% |
| G^+^ | *Streptococcus* | 973 | 92% |
| G^-^ | *Escherichia* | 257 | 66% |
| G^+^ | *Ligilactobacillus* | 360 | 18% |
| G^+^ | *Gemella* | 26 | 3% |
| G^+^ | *Lactococcus* | 1 | 1% |

G: Gram staining

**Table S2: Antibodies information**

|  | Source | Catalog  number | Host  species | Dilution |
| --- | --- | --- | --- | --- |
| 6E10 | Biolegend | 803001 | Mouse | IF/IHC 1:500 |
| ZO-1 | Proteintech | 21773-1-AP | Rabbit | WB 1:1000  IF 1:1000 |
| Claudin-1 | Proteintech | 28674-1-AP | Rabbit | WB 1:1000  IF 1:600 |
| Occludin | Proteintech | 66378-1-Ig | Mouse | WB 1:5000 |
| GAPDH | Proteintech | 60004-1-Ig | Mouse | WB 1:3000 |
| Iba1 | WAKO | 019-19741 | Rabbit | IF 1:600 |
| APP | SIGMA | SAB4300464 | Rabbit | WB 1:1000  IF 1:1000 |
| PS1 | SIGMA | PRS4203 | Rabbit | WB 1:1000  WB 1:1000 |
| BACE1 | Millipore | MAB5308 | Mouse | WB 1:1000 |
| ADAM10 | Millipore | AB19026 | Rabbit | WB 1:1000 |
| IDE | Abcam | ab32216 | Rabbit | WB 1:1000 |
| NEP | Millipore | AB5458 | Rabbit | WB 1:800 |
| LRP1 | Abcam | ab92544 | Rabbit | WB 1:1000 |
| TLR4 | ABclonal | A17436 | Rabbit | WB 1:1000 |
| p-IKKβ | CST | 2697s | Rabbit | WB 1:1000 |
| IKKβ | CST | 8943S | Rabbit | WB 1:1000 |
| p-P65 | CST | 3033s | Rabbit | WB 1:1000 |
| P65  Iba1  CD68  Horseradish enzyme labeled  goat anti-mouse IgG (H+L)  Horseradish enzyme labeled goat anti-rabbit IgG (H+L)  AF488-conjugated anti-rabbit IgG  AF555-conjugated anti-rabbit IgG  AF555-conjugated anti-mouse IgG  AF555-conjugated anti-goat IgG  AF488-conjugated anti-rat IgG | CST  Abcam  Abcam  ZSGB-BIO  ZSGB-BIO  Thermo Fisher  Thermo Fisher  Thermo Fisher  Thermo Fisher  Abcam | 8242  ab5076  ab53444  ZB-2305  ZB-2301  A21206  A31572  A31570  A21432  AB150153 | Rabbit  Goat  Rat  Goat  Goat  Donkey  Donkey  Donkey  Donkey  Donkey | WB 1:1000  IF 1:200  IF 1:500  WB 1:2000  WB 1:2000  IF 1:1000  IF 1:1000  IF 1:1000  IF 1:1000  IF 1:1000 |

**
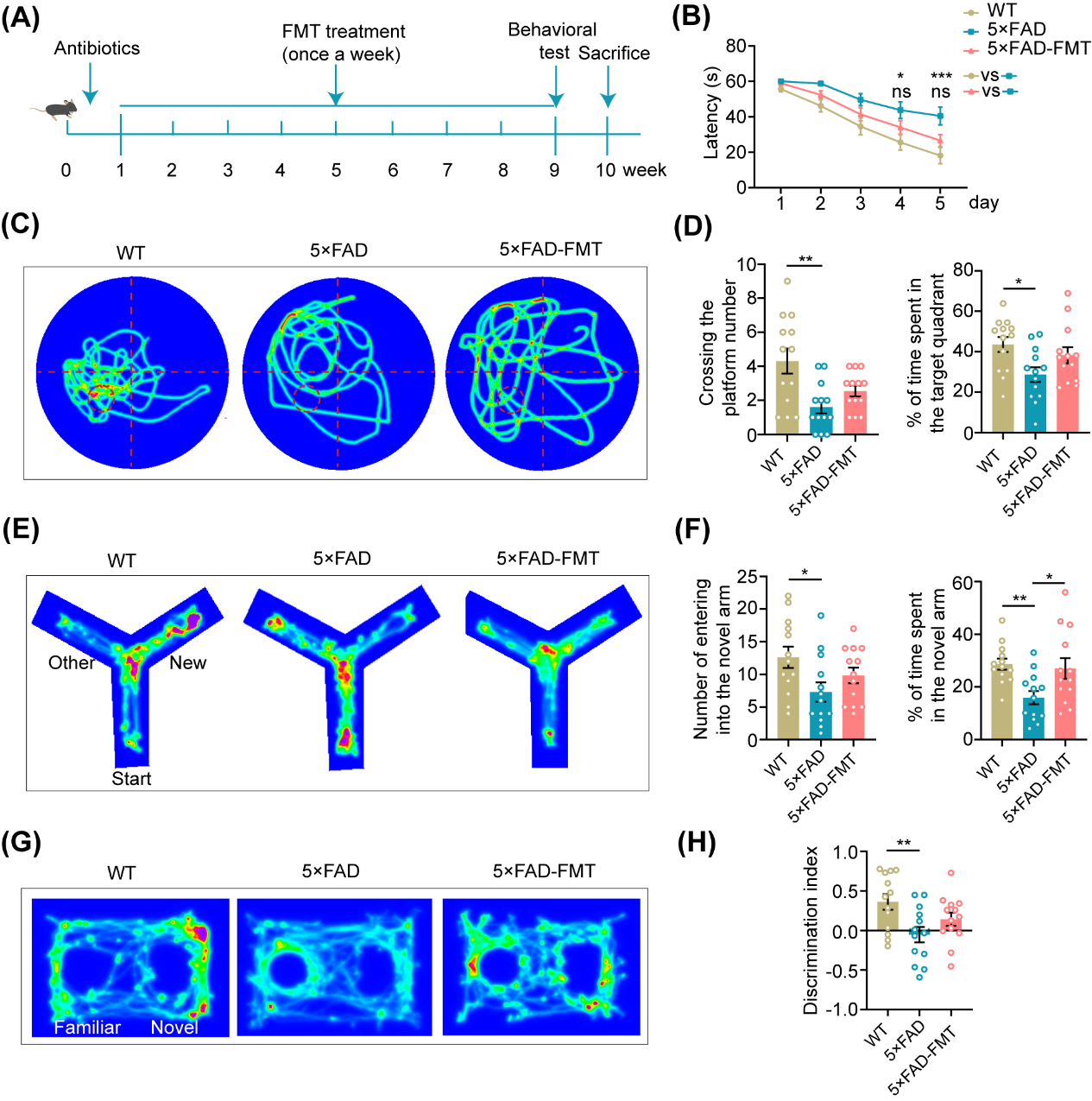
FIGURE S1** **Eight weeks of weekly FMT did not alleviate memory deficits in 5×FAD mice.** **(A)** Schematic representation of the experimental process. Mice underwent antibiotic treatment, weekly FMT for eight consecutive weeks, behavioral tests, and sacrifices. **(B)** Statistical analysis of the mean latency to reach the platform across the Morris water maze training days. **(C)** Representative mouse swim paths on the sixth day (testing phase) of the Morris water maze. **(D)** Quantification of platform crossings and the percentage of time spent in the target quadrant during the Morris water maze test. **(E)** Representative movement traces of mice in the Y-maze test. **(F)** Statistical analysis of the number of entries and the percentage of time spent exploring the novel arm during the Y-maze test phase. **(G)** Movement traces of mice in the novel object recognition test. **(H)** Statistical analysis of the discrimination index in the novel object recognition test. n = 13 per group. Significance was evaluated by repeated measures two-way ANOVA with Tukey's post hoc test (**B**), and all other data were evaluated by one-way ANOVA with Dunnett's post hoc test. **p* < 0.05, ***p* < 0.01, ****p* < 0.001.

**
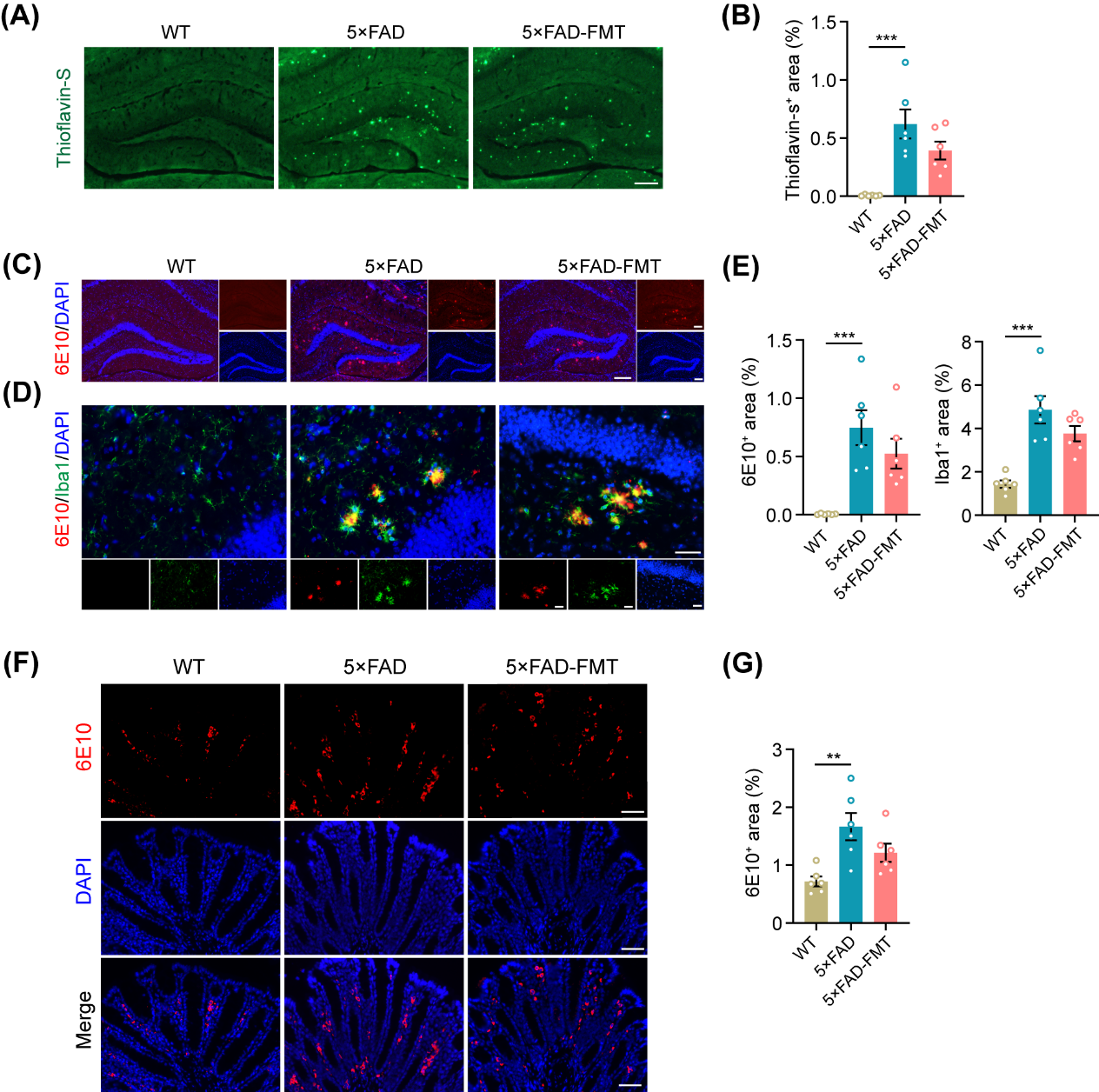
**

**FIGURE S2 Eight weeks of weekly FMT did not reduce hippocampal Aβ deposition, neuroinflammation, or colonic Aβ pathology in 5×FAD mice. (A)** Representative images of Thioflavin-S^+^ plaques in the hippocampus. Scale bar, 200 μm. **(B)** Statistical analysis of the percentage of Thioflavin-S^+^ area in the hippocampus. **(C-D)** Representative immunofluorescence images of 6E10 (Red), Iba1 (Green), and DAPI (Blue) in the hippocampus of each group. Scale bar, 200 μm for (**C**), and 40 μm for (**D**). **(E)** Quantification of the percentage of 6E10^+^ and Iba1^+^ area in the hippocampus. **(F)** Representative immunofluorescence images of 6E10 in the colon. Scale bar, 40 μm. **(G)** Statistical analysis of the percentage of 6E10^+^ area in the colon. n = 6 per group. Statistical significance was assessed using one-way ANOVA with Dunnett's post hoc test. ***p* < 0.01, ***p < 0.001.

**
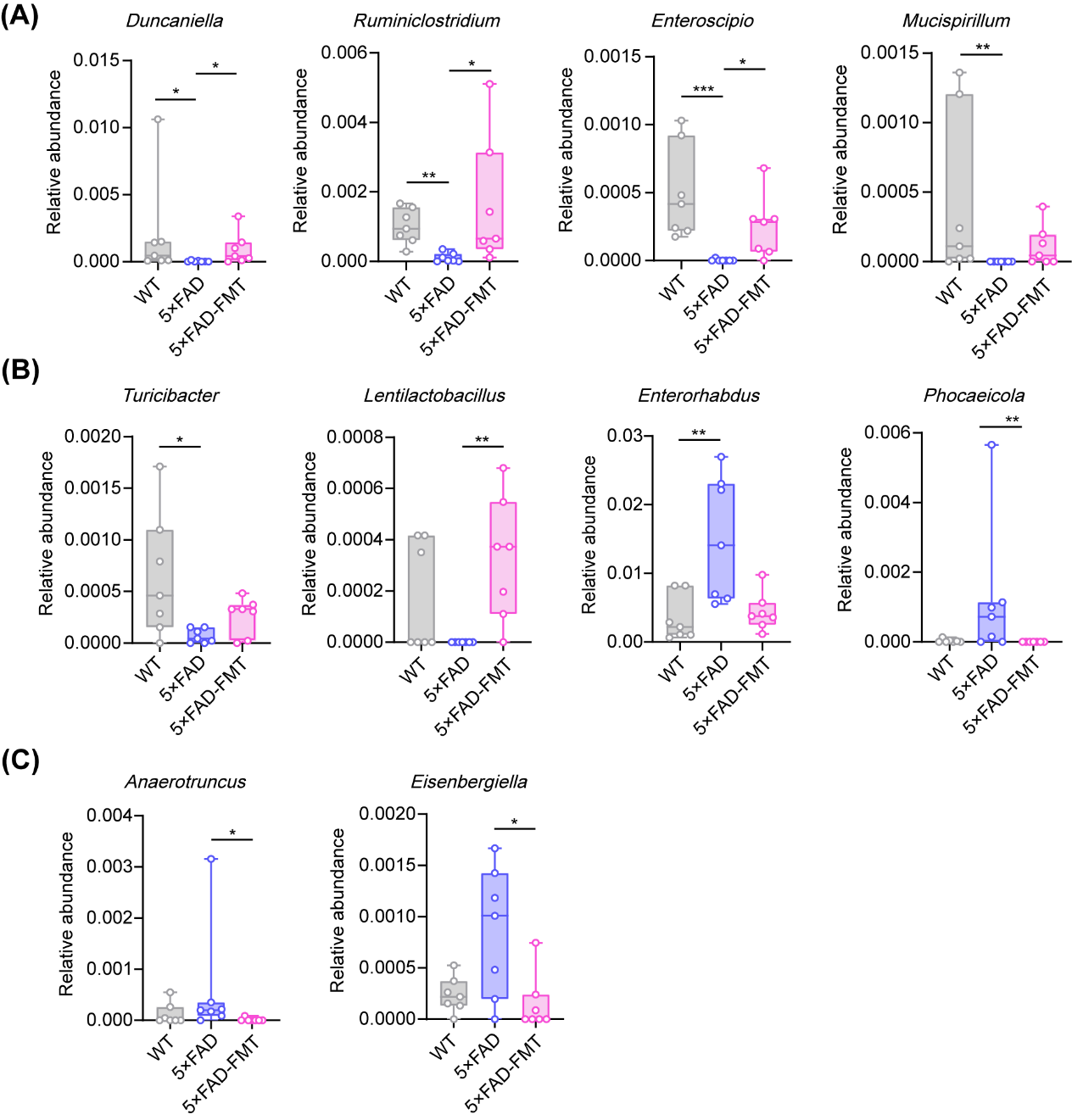
**

**FIGURE S3 Alterations in the gut microbiota composition at the genus level in 5×FAD mice following FMT. (A-C)** Statistical analysis of the relative abundance of *Duncaniella*, *Ruminiclostridium*, *Enteroscipio*, *Mucispirillum*, *Turicibacter*, *Lentilactobacillus*, *Enterorhabdus*, *Phocaeicola*, *Anaerotruncus*, and *Eisenbergiella* among the three groups. n = 7 per group. Statistical significance was assessed using the Kruskal-Wallis test with Dunn's post hoc test for multiple comparisons. **p* < 0.05, ***p* < 0.01, ***p < 0.001.

**
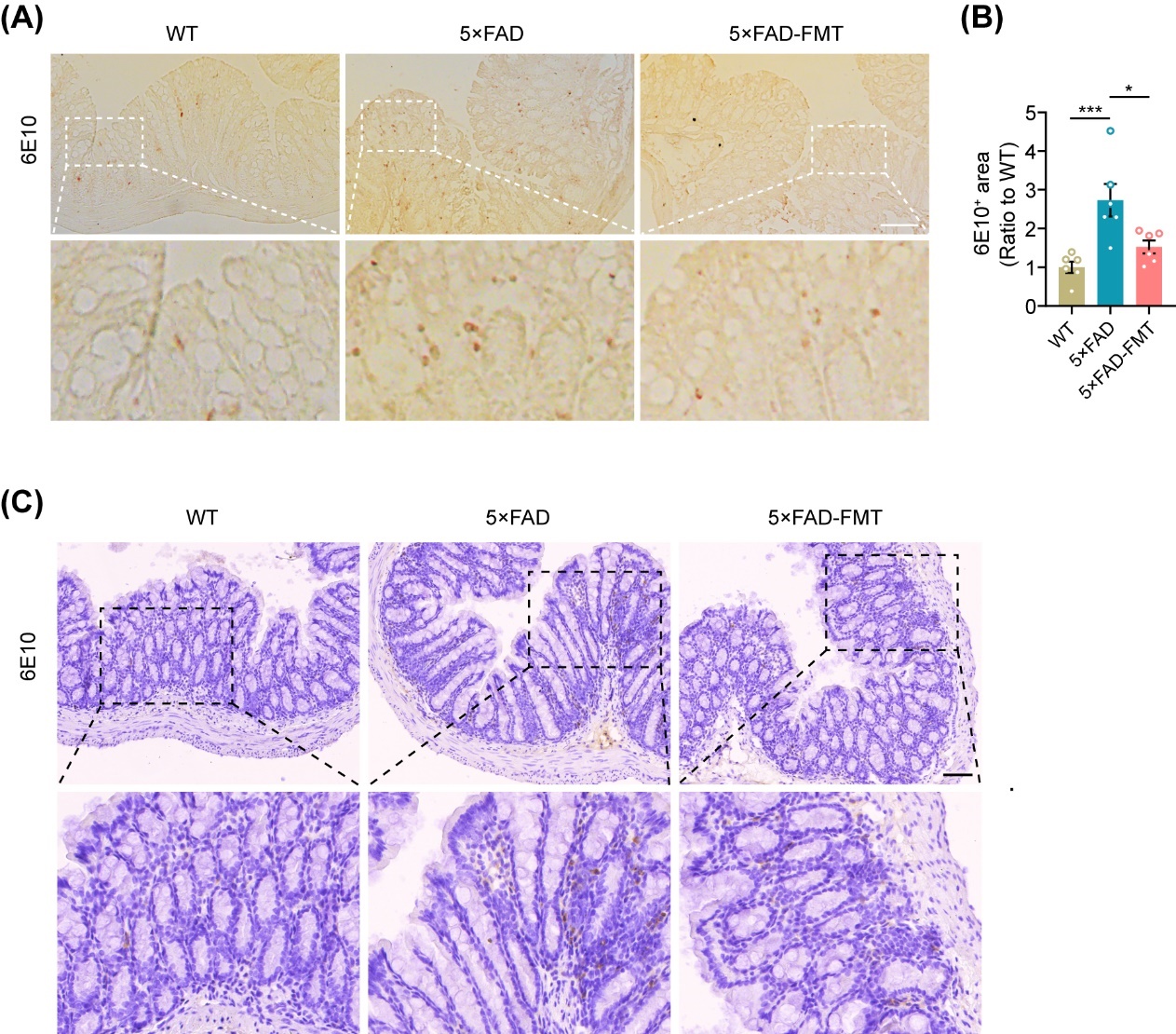
FIGURE S4 FMT-mediated improvement on colonic Aβ pathology in 5×FAD mice. (A)** Representative immunohistochemical images of 6E10 staining in the colon from each group. Scale bar, 100 μm. **(B)** Statistical analysis of the percentage of 6E10^+^ area in the colon. **(C)** Representative images of 6E10 immunohistochemical staining with hematoxylin counterstaining in the colon. Scale bar, 60 μm. n = 6 per group. Statistical significance was evaluated by one-way ANOVA with Dunnett's post hoc test. **p* < 0.05, ****p* < 0.001.


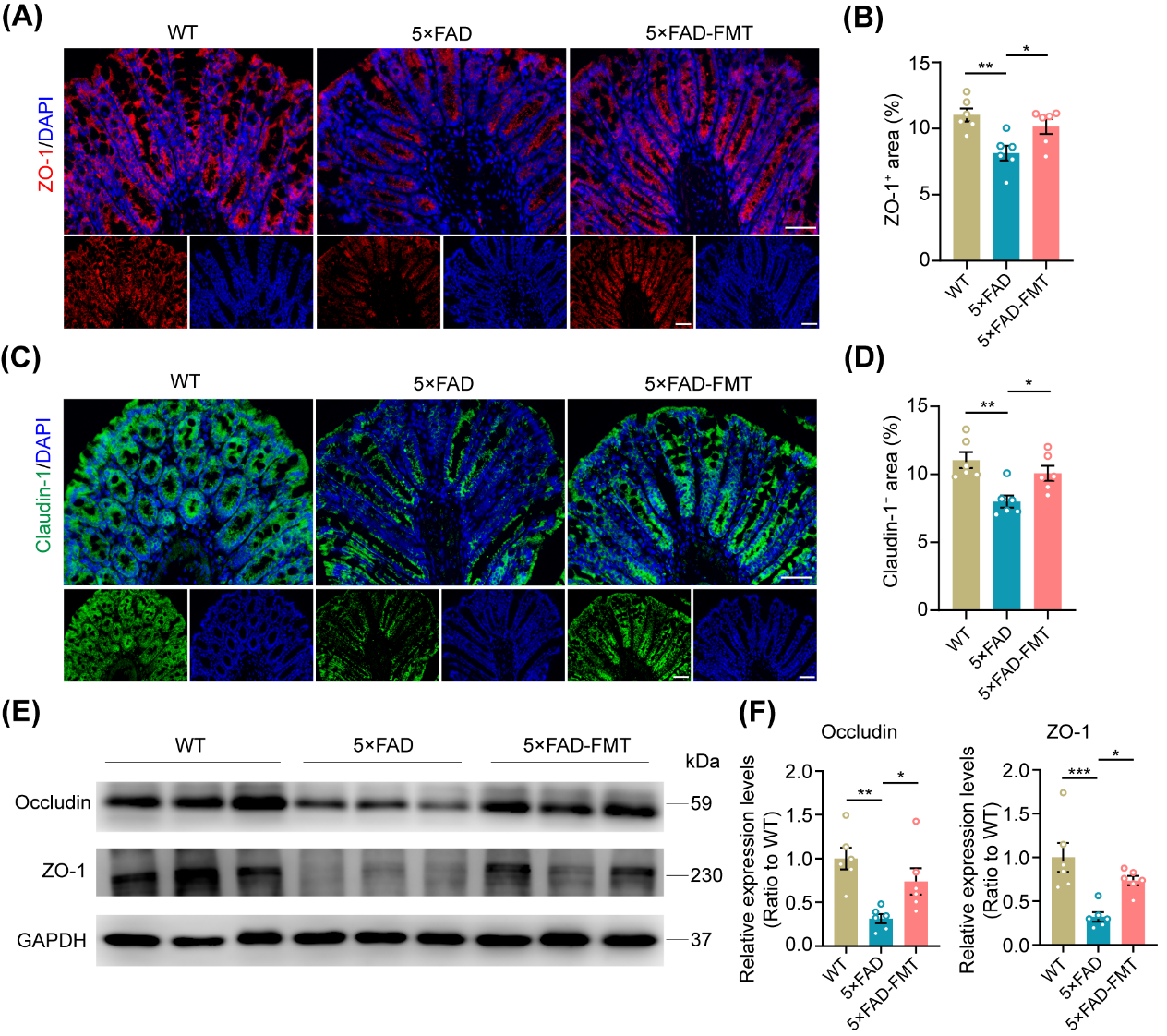


**FIGURE S5 FMT-mediated improvement of intestinal barrier function in 5×FAD mice**. **(A)** Immunofluorescence images of ZO-1 in the colon. Scale bar, 40 μm. **(B)** Percentage of ZO-1^+^ area in the colon. **(C)** Immunofluorescence images of Claudin-1 in the colon. Scale bar, 40 μm. **(D)** Percentage of Claudin-1^+^ area in the colon **(E-F)** Representative Western blot bands and densitometry analysis of Occludin and ZO-1 in the colon of all groups. n = 6 per group. All statistical analyses were performed using one-way ANOVA with Dunnett's post hoc test. **p* < 0.05, ***p* < 0.01, ****p* < 0.001.

**
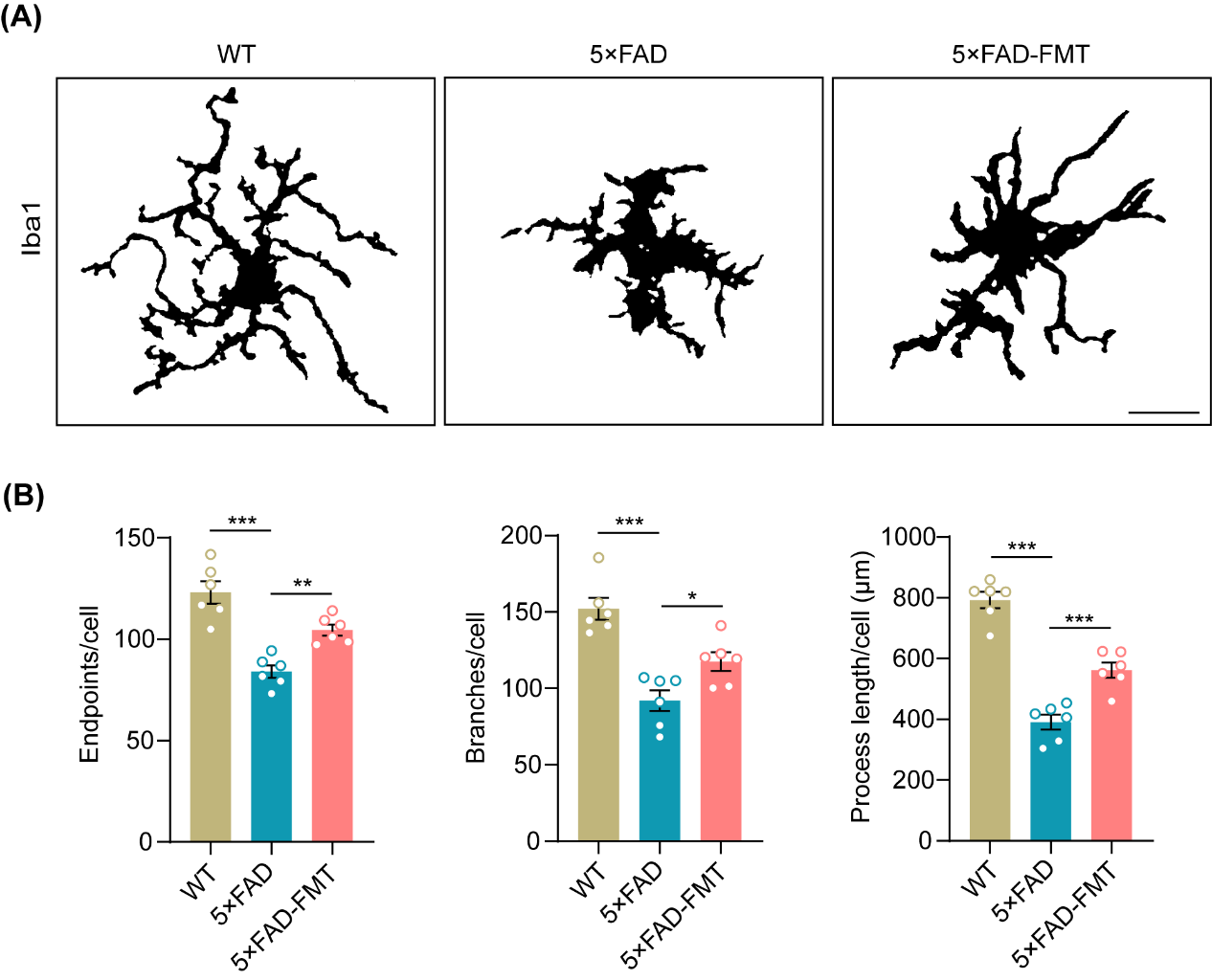
****FIGURE S6 FMT-induced morphological changes in microglia. (A)** Representative binary images of Iba1^+^ microglia. Scale bar, 20 μm. **(B)** Quantitative analysis of the total number of endpoints, branches, and process length per cell. n = 6 per group. Statistical significance was assessed using one-way ANOVA with Dunnett's post hoc test. **p* < 0.05, ***p* < 0.01, ***p < 0.001.

**
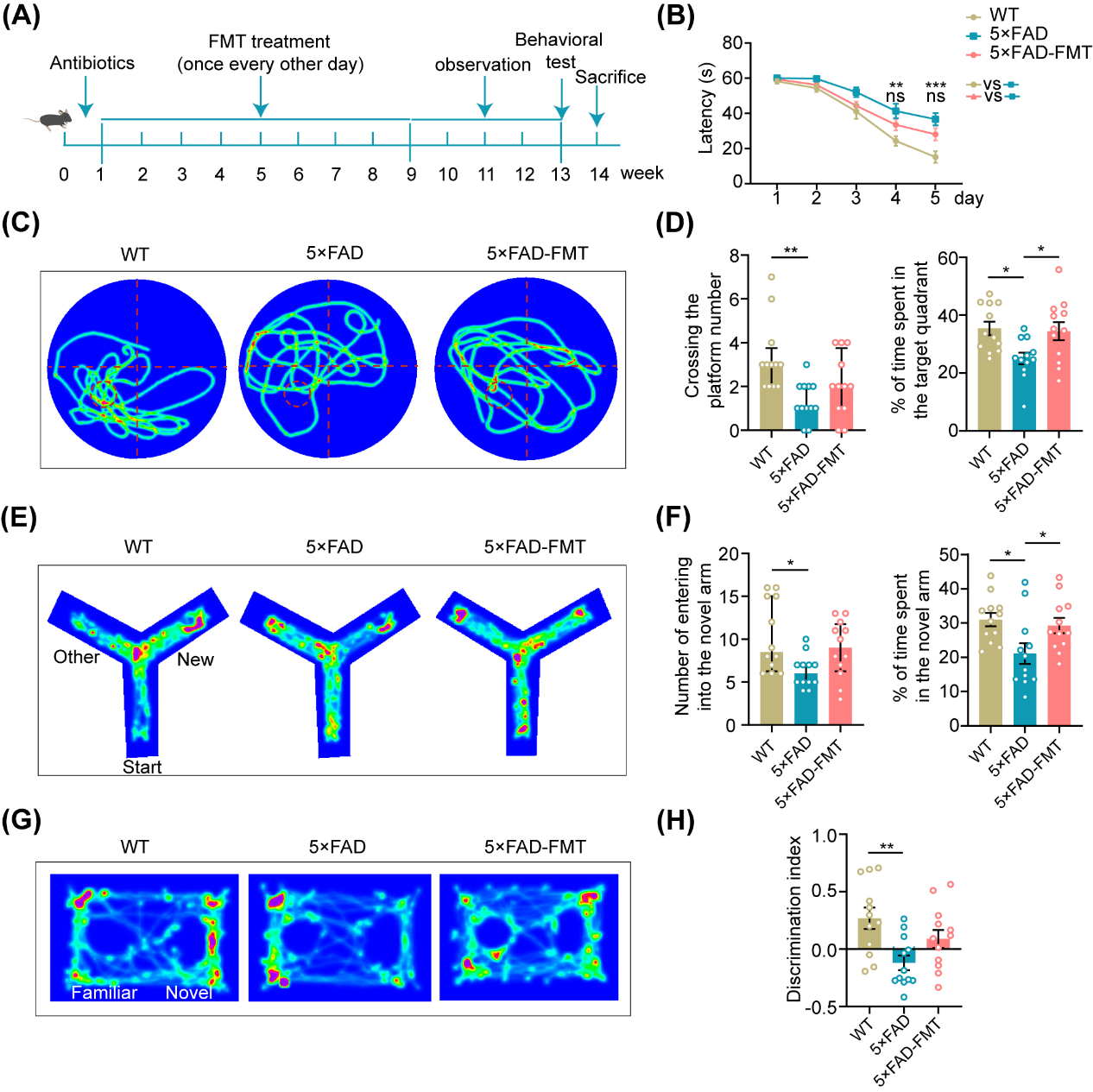
**

**FIGURE S7 The protective effects of FMT on memory impairment in 5×FAD mice did not persist four weeks after discontinuation. (A)** Schematic representation of the experimental design. Mice underwent antibiotic treatment, FMT (administered every other day for 8 weeks), a no-intervention observation period, behavioral testing, and euthanasia. **(B)** Statistical analysis of the mean latency to reach the platform in the Morris water maze during the training phase. **(C)** Swimming paths on the sixth day (probe trial) of the Morris water maze. **(D)** Analysis of the number of platform crossings and the time spent in the target quadrant during the Morris water maze probe trial. **(E)** Movement tracks of mice in the Y-maze test. **(F)** Statistical analysis of the number of entries and the percentage of time spent exploring the novel arm during the Y-maze test phase. **(G)** Movement tracks of mice in the novel object recognition test. **(H)** Discrimination index in the novel object recognition test. n = 12 per group. Significance was evaluated using repeated measures two-way ANOVA with Tukey's post hoc test for the latency data in (**B**), the Kruskal-Wallis test with Dunn's post hoc analysis for the platform crossings in (**D**), and novel arm entries in (**F**), and one-way ANOVA with Dunnett's post hoc test for all other data. **p* < 0.05, ***p* < 0.01, ****p* < 0.001.

**
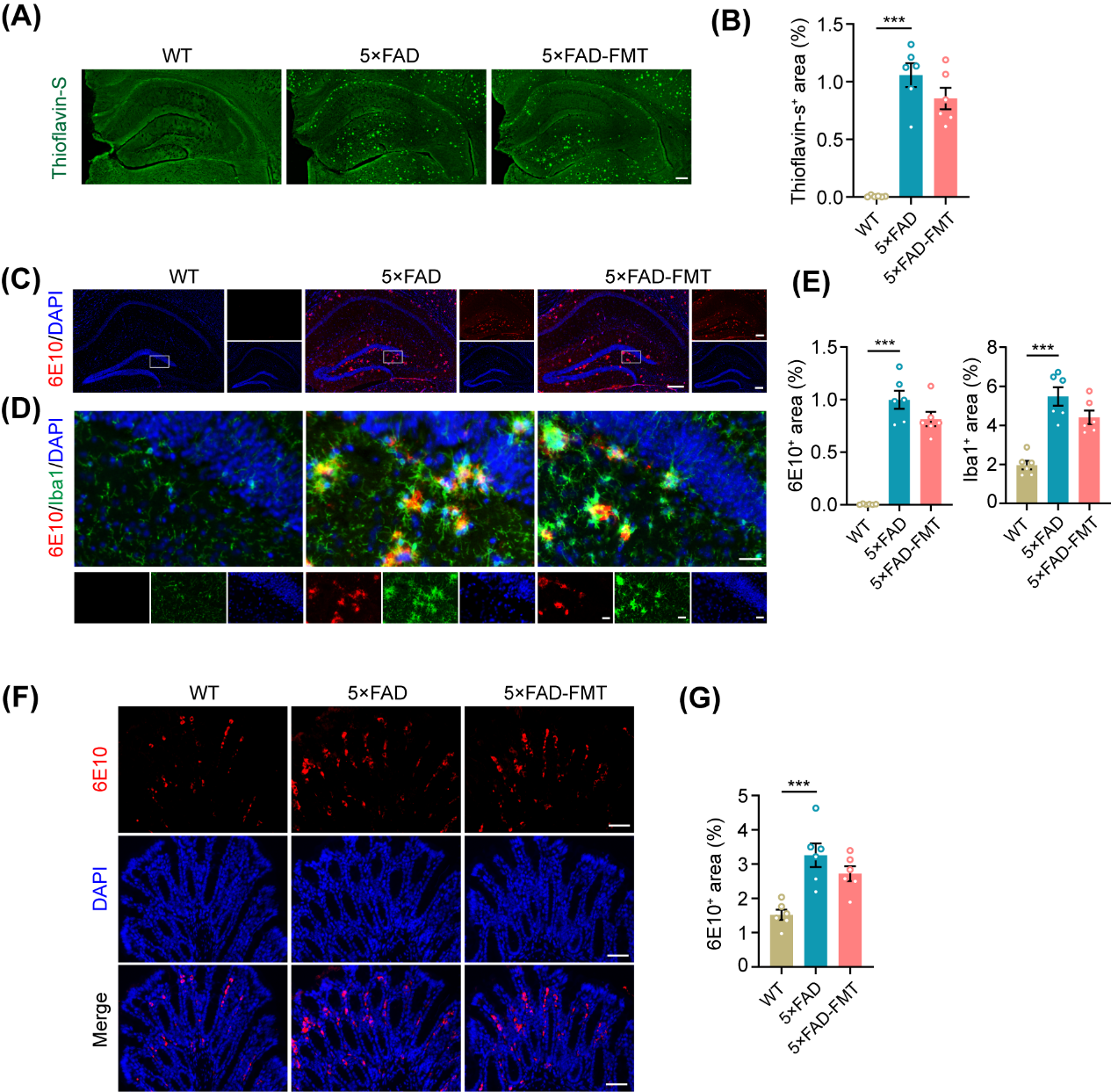
FIGURE S8 The protective effects of FMT on hippocampal Aβ deposition, neuroinflammation, and colonic Aβ pathology in 5×FAD mice** **waned within 4 weeks after the intervention was discontinued. (A)** Representative images of Thioflavin-S staining in the hippocampus. Scale bar, 200 μm. **(B)** Percentage of Thioflavin-S^+^ area in the hippocampus. **(C-D)** Representative immunofluorescence images of 6E10 (Red), Iba1 (Green), and DAPI (Blue) in the hippocampus. Scale bar, 200 μm for (**C**), and 40 μm for (**D**). **(E)** Statistical analysis of the percentage of 6E10^+^ and Iba1^+^ areas in the hippocampus. **(F)** Representative immunofluorescence images of 6E10 in the colon. Scale bar, 40 μm. **(G)** Statistical analysis of the percentage of 6E10^+^ area in the colon. n = 6 per group. Significance was evaluated by one-way ANOVA with Dunnett's post hoc test. ****p* < 0.001.
